# Systematic multi‐level analysis of an organelle proteome reveals new peroxisomal functions

**DOI:** 10.15252/msb.202211186

**Published:** 2022-09-27

**Authors:** Eden Yifrach, Duncan Holbrook‐Smith, Jérôme Bürgi, Alaa Othman, Miriam Eisenstein, Carlo WT van Roermund, Wouter Visser, Asa Tirosh, Markus Rudowitz, Chen Bibi, Shahar Galor, Uri Weill, Amir Fadel, Yoav Peleg, Ralf Erdmann, Hans R Waterham, Ronald J A Wanders, Matthias Wilmanns, Nicola Zamboni, Maya Schuldiner, Einat Zalckvar

**Affiliations:** ^1^ Department of Molecular Genetics The Weizmann Institute of Science Rehovot Israel; ^2^ Institute of Molecular Systems Biology ETH Zurich Zurich Switzerland; ^3^ Hamburg Unit c/o DESY European Molecular Biology Laboratory (EMBL) Hamburg Germany; ^4^ Laboratory Genetic Metabolic Diseases, Department of Clinical Chemistry, Amsterdam Gastroenterology, Endocrinology & Metabolism Amsterdam University Medical Centers – Location AMC Amsterdam The Netherlands; ^5^ Life Sciences Core Facilities (LSCF) The Weizmann Institute of Science Rehovot Israel; ^6^ Department of Systems Biochemistry, Institute of Biochemistry and Pathobiochemistry Ruhr‐University Bochum Bochum Germany; ^7^ University Medical Center Hamburg‐Eppendorf Hamburg Germany

**Keywords:** high‐content screen, high‐resolution imaging, peroxisome, protein targeting, *Saccharomyces cerevisiae*, Organelles

## Abstract

Seventy years following the discovery of peroxisomes, their complete proteome, the peroxi‐ome, remains undefined. Uncovering the peroxi‐ome is crucial for understanding peroxisomal activities and cellular metabolism. We used high‐content microscopy to uncover peroxisomal proteins in the model eukaryote – *Saccharomyces cerevisiae*. This strategy enabled us to expand the known peroxi‐ome by ~40% and paved the way for performing systematic, whole‐organellar proteome assays. By characterizing the sub‐organellar localization and protein targeting dependencies into the organelle, we unveiled non‐canonical targeting routes. Metabolomic analysis of the peroxi‐ome revealed the role of several newly identified resident enzymes. Importantly, we found a regulatory role of peroxisomes during gluconeogenesis, which is fundamental for understanding cellular metabolism. With the current recognition that peroxisomes play a crucial part in organismal physiology, our approach lays the foundation for deep characterization of peroxisome function in health and disease.

## Graphical abstract

High‐content imaging analysis uncovers the peroxisomal proteome of *Saccharomyces cerevisiae*. Systematic, whole‐organellar proteome assays reveal new players in peroxisome organization, targeting, and function.

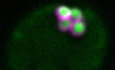

## Introduction

All eukaryotic cells, from yeast to humans, compartmentalize various functions into membrane‐enclosed organelles. One such organelle, the peroxisome, is crucial for human health and survival as it hosts essential metabolic enzymes (Waterham *et al*, 2016). Despite its importance to organismal health and cellular metabolism, the full repertoire of proteins that reside within peroxisomes had not been fully uncovered.

One substantial leap that fueled the scientific community's interest in other metabolic organelles such as the mitochondria was the formation of its protein compendium, the MitoCarta (Pagliarini *et al*, 2008). To put peroxisomes into the limelight, and expose the complete variety of peroxisomal functions, we set out to uncover the peroxisomal proteome, the peroxi‐ome.

The discovery of the entire peroxi‐ome is important not only in the diagnosis and treatment of patients suffering from peroxisomal diseases but much more broadly in the study of viral infection and immunity (Cook *et al*, 2019), malignant transformation (Kim, 2020), aging (Titorenko & Terlecky, 2011; Pascual‐Ahuir *et al*, 2017), and neurodegenerative diseases (Zarrouk *et al*, 2020) – all of which have now clearly demonstrated a role for peroxisomes in their progression.

However, discovering the complete peroxi‐ome is a challenging task – peroxisomes are small and physically attach to multiple other organelles via contact sites (Shai *et al*, 2016; Valm *et al*, 2017; Castro *et al*, 2018; Schrader *et al*, 2020). Peroxisomes also change dramatically in response to cell state or environmental conditions (Smith & Aitchison, 2013).

Various systematic studies were previously used to discover peroxisomal proteins. Most efforts relied on either mass‐spectrometry‐based analysis of peptides in subcellular fractions, or on sequence‐based strategies to detect proteins with a potential canonical Peroxisomal Targeting Signal 1 or 2 (PTS1 or PTS2; Schrader & Fahimi, 2008), which are known to be recognized by peroxisomal targeting factors (Walter & Erdmann, 2019). However, both of these approaches have limitations such as the difficulty in detecting low‐abundance and conditionally expressed proteins by subcellular fractionations, or the inability to find non‐canonically targeted proteins by sequence‐based approaches. Based on these efforts, as well as low‐throughput protein‐specific studies, we have recently curated comprehensive lists of peroxisome proteomes in humans, mice (Yifrach *et al*, 2018), and *Saccharomyces cerevisiae* (from herein called yeast; Yifrach *et al*, 2016). These efforts highlighted that proteins responsible for known peroxisomal activities (Grunau *et al*, 2009; Antonenkov & Hiltunen, 2012) have not yet been described and suggested that new approaches are necessary to identify more peroxisomal proteins.

We, therefore, decided to take a complementary approach and sought to map the peroxi‐ome by performing a high‐content screen on fluorescently tagged yeast proteins. Here we report the identification of 33 peroxisomal proteins, which together with the known ones make the most complete inventory of the peroxi‐ome to date, containing 115 proteins in total (Dataset EV1). Having a comprehensive view of the peroxi‐ome enabled us to create a new strategy to characterize peroxisomal activities – one that relies on a systematic analysis of the entire peroxi‐ome at various functional levels. For example, by systematically studying the sub‐organellar localization and targeting mode of the entire organellar proteome, we exposed non‐canonical targeting dependencies on the main targeting factor, Pex5. By proteome‐wide analysis of the metabolomic profiles of peroxisomal mutants, we revealed unexpected metabolic functions for three enzymes in peroxisomes. Importantly, we identified a novel mechanism by which peroxisomes regulate gluconeogenesis, a process that generates sugars from non‐carbohydrate substrates. This finding exposes an unexpected link between peroxisomal activity and gluconeogenesis that goes beyond the provision of building blocks following fatty acid degradation. More broadly, the identification of tens of peroxisomal proteins paves the way for additional exciting discoveries regarding peroxisome function and regulation and introduces a more holistic view on this important, yet understudied organelle.

## Results

### A high‐content screen maps the peroxi‐ome

To make the peroxi‐ome more complete, we performed a high‐throughput microscopic screen on a recently made full‐genome yeast collection, harboring a green fluorescent protein (GFP) tag fused to the amino terminus (N′) of each yeast protein (Weill *et al*, 2018). This strategy has several advantages: (i) The cells are analyzed by imaging keeping all cellular structures intact. This is especially important for the proper identification of proteins that are localized to several compartments and could be regarded as contaminants in mass‐spectrometry‐based analysis of enriched peroxisomal fractions as they may not be enriched in these fractions alone, (ii) The method is unbiased and does not rely on any prior knowledge on the protein sequence and hence can uncover proteins that target to peroxisomes without classical targeting motifs, (iii) Expression is regulated under a constitutive *NOP1* promoter, which enables us to detect the localization of low abundance and conditionally expressed proteins, (iv) Having a GFP tag on the N′ of each protein allows proper targeting of all known proteins with either a C′ PTS1 or an N′ PTS2 motif, as we have previously shown (Yofe *et al*, 2016), and (v) We have previously performed a pilot experiment and verified that this methodology works well in identifying more peroxisomal proteins (Yifrach *et al*, 2016).

**Figure 1:**
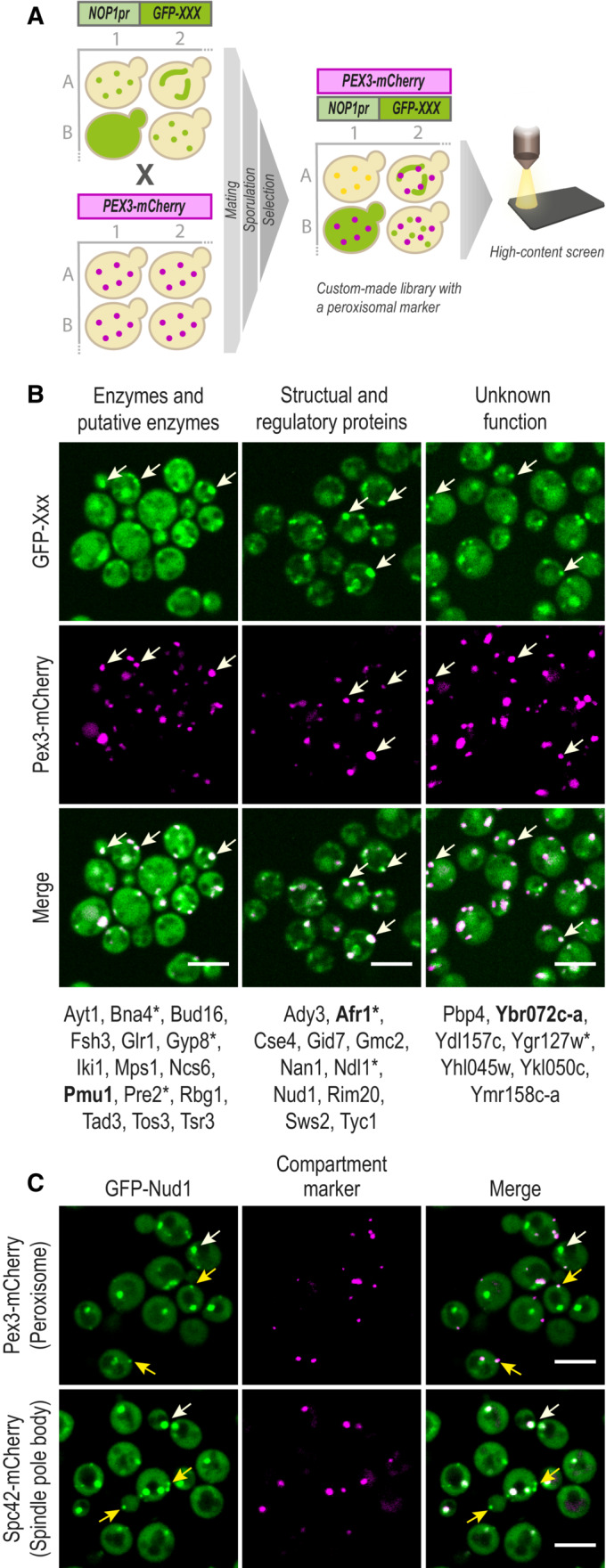
A high‐content screen maps the peroxi‐ome A peroxisomal marker Pex3‐mCherry was genomically integrated into a yeast N′ GFP collection by utilizing an automated mating procedure. Then, high‐throughput fluorescence microscopy was applied to identify proteins that co‐localize with the peroxisomal marker. Only proteins that passed three validation steps (manual retagging, PCR, and western blot (Appendix Fig S1)) were determined as newly identified peroxisomal proteins.The validated proteins were divided into groups to represent their variety of functional annotations: enzymatic, structural, or unknown activity. In bold are the proteins whose images are presented. All proteins were tested for their localization when their encoding genes are expressed under their native promoter (Dataset EV1); asterisks mark the ones that could be visualized in peroxisomes.Protein localization image analysis demonstrated that about half of the newly identified proteins are dually localized to peroxisomes and other compartments. Presented is GFP‐Nud1, which co‐localizes with both a spindle‐pole body marker (white arrows) and a peroxisomal marker (yellow arrows). A full analysis of dual‐localization is shown in Appendix Fig S2 and Dataset EV1. Data information: For all micrographs, a single focal plane is shown. The scale bar is 5 μm.

To unequivocally identify peroxisomal structures, we integrated a peroxisomal marker, Pex3‐mCherry, into the entire yeast library using an automated mating procedure (Tong & Boone, 2006; Cohen & Schuldiner, 2011) and screened the new, custom‐made library, containing both the N′ GFP tagged proteins and the peroxisomal marker using an automated fluorescence microscope (Fig 1A). To sensitize the screen, we grew the yeast on the fatty acid oleate as a sole carbon source, a condition that makes peroxisomes larger, more numerous, and essential for yeast survival. Moreover, some proteins, although expressed in glucose, are localized to peroxisomes only in oleate‐containing media, as we have previously shown (Yifrach *et al*, 2016). To ensure that we are not missing proteins that co‐localize to peroxisomes only in glucose, we performed an additional screen in glucose‐containing medium only for 280 strains that were annotated to have a punctate localization in glucose in the original N′ GFP library (Weill *et al*, 2018).

Following manual analysis of all images, we found 50 N′ GFP‐tagged proteins that co‐localized with the peroxisomal marker and were not previously reported as peroxisomal proteins. We performed several verification steps including manual re‐tagging with GFP of all 50 proteins, ascertaining correct genomic integration using PCR, re‐imaging, and western blot analysis (Appendix Fig S1). This stringent filtering narrowed down the initial list to 33 verified newly identified peroxisomal proteins (Dataset EV1). The newly identified proteins (from now on called hits) expand the present protein count of peroxisomes by ~40%. These proteins include enzymes and putative enzymes, structural and regulatory proteins, and uncharacterized proteins of unknown function (Fig 1B). We re‐imaged all hits when their respective genes were expressed under their native promoter (Dataset EV1) and found that more than 60% of the proteins could not be visualized above background, which may explain their absence in previous proteomics‐based approaches. For six proteins, their peroxisomal localization was still visual, verifying that the constitutive expression only enhanced the signal and did not change the proteins' endogenous localization (Fig 1B, marked with asterisks). For five proteins that were not visualized in peroxisomes when expressed under their native promoter, it may still be that their peroxisomal localization is not visible above background autofluorescence in these conditions, as we have previously shown for a *bone fide* peroxisomal protein that could be visualized in peroxisomes only when overexpressed (Shai *et al*, 2018).

Interestingly, ~50% of the proteins in our list are dually localized to peroxisomes and other compartments such as mitochondria, nucleolus, and bud‐neck (Fig 1C, Dataset EV1, and Appendix Fig S2). We considered proteins as dual localized only when their tagged protein was visualized in at least one localization similar to its annotation in “SGD cellular compartment” database. The proteins' dual localization may explain why they were not previously identified as peroxisomal proteins and demonstrate the advantage of using an imaging‐based approach. For example, Nud1 is a core component of the spindle pole body (SPB) outer plaque. We show that, as expected, GFP‐Nud1 co‐localizes with an SPB marker, but that it has an additional, secondary, localization to peroxisomes (Fig 1C).

Overall, our approach uncovered tens of proteins that, by fluorescence microscopy resolution, seem to associate with peroxisomes, although they were not assigned as such before. This puts forward the most comprehensive peroxi‐ome list, which can be now used to study different aspects of peroxisome biology using systematic, whole‐proteome technologies, to discover new concepts in peroxisome biology.

### High‐resolution imaging reveals the sub‐organellar distribution of the peroxi‐ome

To further characterize the peroxi‐ome, we wanted to uncover the sub‐organellar distribution of peroxisomal proteins systematically. Regular fluorescence microscopes are limited to a resolution of ~250 nm. This resolution is not sufficient for ascertaining co‐localization between small cellular structures nor for detecting the sub‐organellar distribution of proteins in most organelles. Therefore, we exploited a long‐established observation in which deletion of the peroxisomal membrane protein Pex11 and incubation of cells in oleate‐containing media causes the enlargement of peroxisomes (Erdmann & Blobel, 1995). We noticed that the C terminal (C′) tagging of Pex11 causes the same phenomenon. Combining the genetic expansion with high‐resolution microscopy provided us with a sufficient spatial resolution to differentiate between membrane‐ and matrix‐localized organellar proteins.

**Figure 2:**
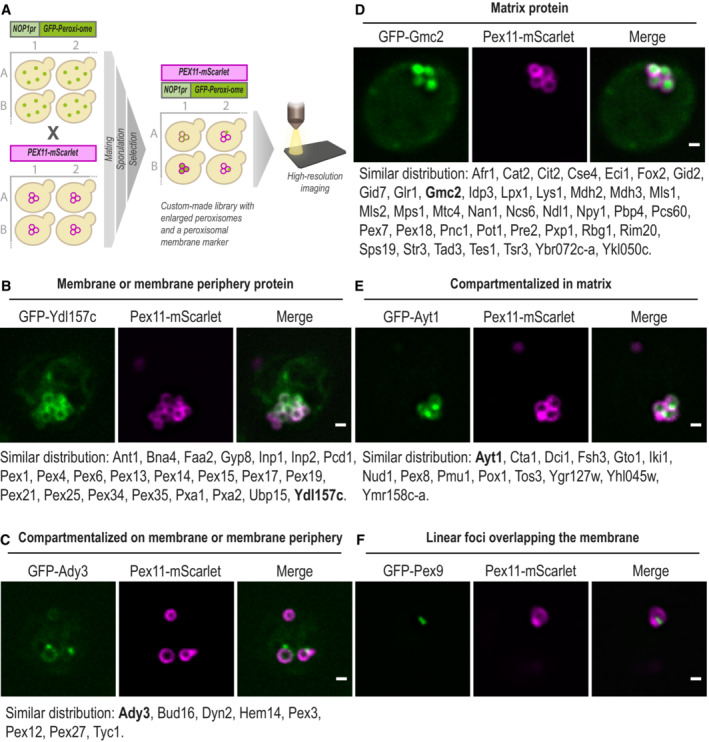
High‐resolution imaging reveals the sub‐organellar distribution of the peroxi‐ome The sub‐peroxisomal localization of each peroxi‐ome protein was captured by high‐resolution imaging and was enabled by genomic and metabolic enlargement of peroxisomes. Enlargement was induced by the integration of the C′ tagged form of the peroxisomal‐membrane protein Pex11 into a yeast N′ GFP collection using an automated mating procedure and upon 20 h of incubation in oleate‐containing media.GFP‐Ydl157c represents a protein that distributes homogeneously on the peroxisomal membrane or membrane periphery.GFP‐Ady3 represents a protein that compartmentalizes on the peroxisomal membrane or membrane periphery.GFP‐Gmc2 represents a protein that distributes homogeneously in the peroxisomal matrix.GFP‐Ayt1 represents a protein that compartmentalizes in the matrix.GFP‐Pex9 was the only protein that showed linear foci overlapping the peroxisomal membrane. Data information: For each category, proteins with similar distributions are listed below the micrographs, when the protein in bold is the one presented. For all micrographs, a single focal plane is shown. The scale bar is 500 nm.

Hence, we applied an automated mating procedure to integrate Pex11‐mScarlet into a yeast collection where each strain expressed one peroxisomal protein fused at its N′ to GFP and imaged all strains by high‐resolution microscopy following growth in oleate‐containing media (Fig 2A).

Analysis of the entire peroxi‐ome distribution revealed five different sub‐peroxisomal localizations. (i) Distributed homogeneously on the peroxisomal membrane or membrane periphery, for example, Ydl157c (Fig 2B), (ii) Compartmentalized on the membrane or the membrane periphery, such as Ady3 (Fig 2C), (iii) Homogeneously distributed in the peroxisomal matrix, like Gmc2 (Fig 2D), (iv) Compartmentalized within the matrix, such as Ayt1 (Fig 2E), and (v) Distributed as linear foci overlapping the membrane, seen only for a single protein, Pex9 (Fig 2F).

Our microscopic results (summarized in Dataset EV1) go hand in hand with previous biochemical data curated for many known peroxisomal proteins and with trans‐membrane domain (TMD) predictions made by systematic topology analysis within TopologYeast (Weill *et al*, 2019). Importantly, we reveal the distribution of all the newly identified peroxisomal proteins and unveil the sub‐organellar localization of several known peroxisomal proteins. For example, some proteins, such as Pex3, Pex12, Pex27, and Hem14, are not evenly distributed but rather seem to compartmentalize on specific sites of the membrane. While Pex3 was previously shown to compartmentalize on the membrane and form contact sites with the plasma membrane (Hulmes *et al*, 2020) and the vacuole (Wu *et al*, 2019), the other proteins were never suggested to have such a distribution. While it may be that some compartmentalization was due to aggregation, it will be interesting to further study whether this distribution is due to their function in membrane contact sites. Overall, exploring which membrane or matrix peroxisomal proteins compartmentalize with which may shed light on potential functional complexes and suggest new roles for peroxisomal proteins.

Furthermore, a unique sub‐peroxisomal distribution was observed for Pex9, a conditional targeting factor that brings a subset of PTS1 proteins into the matrix (Effelsberg *et al*, 2016; Yifrach *et al*, 2016; Fig 2F). It was previously shown that each of the constitutive targeting factors, Pex5 and Pex7, form an import pore together with the docking protein Pex14 to insert folded, and even oligomerized, proteins through the peroxisomal membrane into the matrix (Montilla‐Martinez *et al*, 2015). Our microscopic results, together with the recent finding that Pex9 requires the same set of peroxisomal membrane proteins to mediate the protein import (Rudowitz *et al*, 2020), suggest that it can form a pore with the docking complex on the membrane, which is seen as linear foci overlapping the membrane.

Overall, our sub‐organellar analysis not only confirmed that all the newly identified proteins are indeed intimately connected to peroxisomes, but it also provided a glimpse into the inner‐peroxisomal protein organization and lays the foundations for building a more detailed map of peroxisome function.

### Functional mapping of targeting dependencies for matrix proteins reveals multiple non‐canonical Pex5 substrates

**Figure 3:**
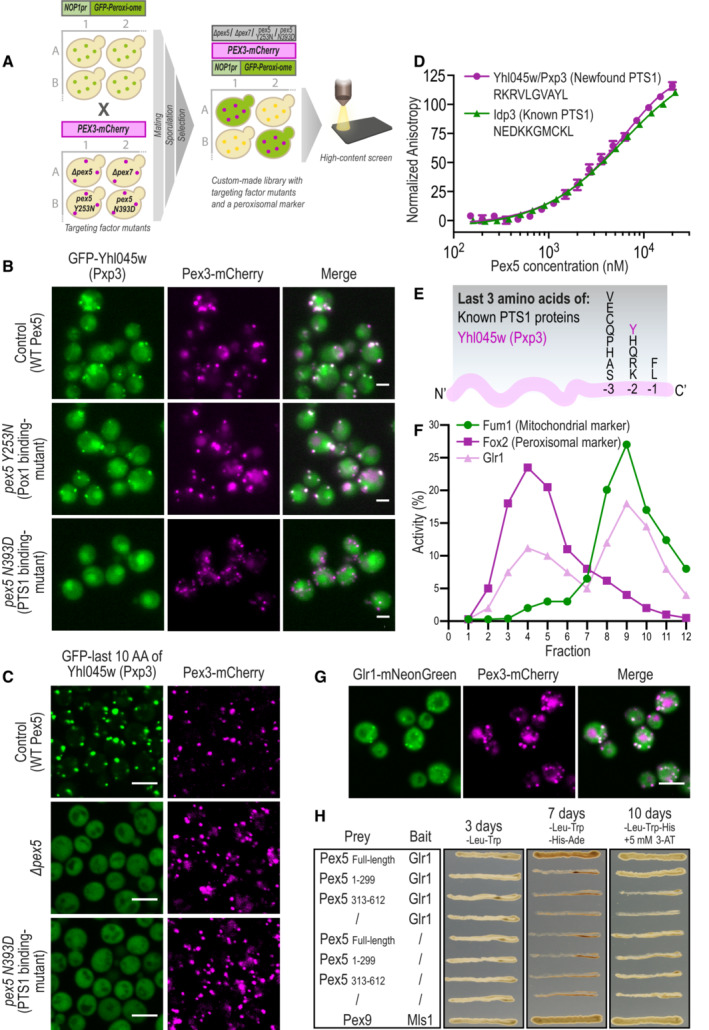
Functional mapping of targeting dependencies for matrix proteins reveals multiple non‐canonical Pex5 substrates Targeting dependencies of the newly identified peroxisomal matrix proteins were uncovered using a high‐content screen. Targeting factor deletions (*∆pex5* or *∆pex7*) or point mutations (*PEX5*_Y253N_ weakens the interaction with Pox1, a non‐PTS1 protein, and *PEX5*_N393D_ weakens the interaction with PTS1 proteins), were used to examine the effects on peroxisomal localization of each newly identified protein. A strain carrying a Hygromycin selection cassette in an inert locus was used as a control for no modification in peroxisomal genes.Matrix proteins targeting was dependent on the PTS1 pathway of the Pex5 targeting factor (full analysis in Dataset EV1). Presented is GFP‐Yhl045w, which was not targeted to peroxisomes upon a point mutation in the PTS1‐binding domain of Pex5 (*PEX5*_N393D_).The peroxisomal targeting ability of the predicted motifs was examined by fusing the last 10 amino acids of each protein to the C′ of GFP, integrating the construct into an inert locus in the yeast genome, and imaging. While most motifs were unable to target GFP to peroxisomes (Appendix Fig S4B), the Yhl045w (Pxp3) motif was sufficient to target GFP to peroxisomes, in a Pex5 PTS1‐binding site‐dependent manner.A fluorescence anisotropy experiment demonstrated direct binding of the Pxp3 motif with purified Pex5 protein (K_d_ = 9.5 ± 2.9 μM), in strength similar to the binding of the known PTS1 motif from Idp3 (K_d_ = 5.5 ± 0.5 μM). The plots represent the mean of three independent experiments, and the error bars represent the standard deviation.The consensus sequence of PTS1 motifs in yeast is now extended with the newly identified, unique, residue (Tyrosine, Y) at position −2 of Pxp3.Subcellular fractionations followed by enzymatic activity assays show that untagged, native, Glr1 is active in peroxisomes, in addition to its known activity in mitochondria. Fum1 and Fox2 were used as markers for mitochondrial, and peroxisomal fractions, respectively.Glr1 C′ was fused to mNeonGreen and its peroxisomal localization was analyzed by fluorescence microscopy, demonstrating that Glr1 does not rely on a free C′ for targeting to peroxisomes hence it does not contain a PTS1 motif.Yeast‐2‐hybrid assay shows Glr1 interacts *in vivo* with both Pex5 full‐length and Pex5 N′ domain. Data information: For all micrographs, a single focal plane is shown. The scale bar is 5 μm.

Following our sub‐organellar analysis, it was clear that most peroxisomal proteins are localized to the matrix; however, many of them do not contain a canonical targeting sequence that is recognized by one of the two main targeting factors, Pex5 and Pex7. Therefore, we wanted to systematically map the targeting pathways that these proteins utilize. To do this we visualized each of the GFP‐tagged peroxisomal proteins on the background of either *PEX5* or *PEX7* deletions (Fig 3A). Known Pex5 or Pex7 cargo proteins served as positive controls to ascertain that we can clearly distinguish specific cargos by this strategy (Appendix Fig S3A). Interestingly, all newly identified matrix proteins depended on *PEX5*, but not on *PEX7*, for their peroxisomal localization (Dataset EV1 and example in Appendix Fig S3B). This may either suggest that Pex7 is indeed quite restricted in its client list. Alternatively, since there are examples of PTS2 proteins whose peroxisomal targeting is (Cartwright *et al*, 2000), or is not (Yofe *et al*, 2016), affected when N′ GFP‐tagged, we cannot rule out that the N′ tagging hinders the targeting of yet unidentified PTS2 proteins. Sequence analysis of the newly identified matrix proteins showed that only six of them were previously predicted to contain the Pex5 targeting signal, PTS1 (Notzel *et al*, 2016). Our work now identifies them as peroxisomal and suggests that this signal is indeed their functional targeting motif.

However, it still remained unclear how the 22 proteins that depend on *PEX5*, but who seemingly do not contain a PTS1 motif, target to peroxisomes. We hypothesized several options: (i) That they have a non‐canonical PTS1 that can still bind to Pex5. (ii) That they do not contain a PTS1, but can “piggyback” on a partner protein that contains a PTS1, as has been previously shown for Mdh2 (Gabay‐Maskit *et al*, 2020). (iii) That they bind Pex5 on a different interface (PTS1‐independent), as shown for a few peroxisomal matrix proteins, the most characterized of which is Pox1 (van der Klei & Veenhuis, 2006; Rymer *et al*, 2018; Kempiński *et al*, 2020).

To unravel which of the above options is relevant for each protein, we first systematically dissected which Pex5 interface they rely on, the PTS1‐ or Pox1‐binding interfaces. To do this we created two strains each with a point mutation in the *PEX5* gene. The first, N393D, is in the PTS1‐binding pocket and weakens the interaction of Pex5 with PTS1 proteins (Klein *et al*, 2002). The second, Y253N, weakens the interaction of Pex5 with Pox1 and possibly other proteins that interact with Pex5 on the same interaction surface (Klein *et al*, 2002). Using known cargos we validated that these mutations allow us to microscopically differentiate between the two binding options (Appendix Fig S3C and D). We introduced the two mutant forms of *PEX5* into the N′ GFP‐tagged peroxi‐ome strain collection (Fig 3A). Surprisingly, we found that the targeting of all 22 novel matrix proteins depends on the PTS1 binding site of Pex5 (Dataset EV1 and an example in Fig 3B). Therefore, we hypothesized that the identified matrix proteins either have a new type of PTS1 or that they piggyback on a PTS1 protein.

To explore potential new PTS1 motifs, we used molecular dynamics (MD) simulations of Pex5/peptide complexes. In short, we used the available experimental structures of human Pex5/cargo complexes (Stanley *et al*, 2006; Fodor *et al*, 2012) to construct complexes of the previously modeled yeast Pex5 PTS1‐binding domain (Gabay‐Maskit *et al*, 2020), with short peptides of six C′ amino acids from known and newly identified, putative, cargo proteins. The constancy of the peptide backbone H‐bonds throughout 180 nanosecond MD trajectories was used to estimate binding stability (Dataset EV2). For the 22 known cargos of Pex5 that have a canonical PTS1, we found that the most stable H‐bonds are those formed by the backbone atoms of peptide residue −1 (most C′) and residue −3. We used these to provide a measure for identifying likely peptide cargos (Appendix Fig S4A box plot and Dataset EV2). We applied the ranges of average stabilities for positions −1 and −3 of the known Pex5 cargos to test the MD trajectories of 12 of the newly identified proteins, six of them previously predicted by sequence to be Pex5 cargos (Notzel *et al*, 2016), and six that were not previously predicted but had similarities to PTS1 sequences (Dataset EV3). Our results predict that some of them can potentially bind to Pex5, interacting with the PTS1 binding pocket at a level similar to that of the 22 known PTS1 cargos.

To experimentally verify the binding predictions, we fused several of the newly predicted motifs to GFP, genomically integrated them into the yeast genome at a locus not affecting cell growth, and tested whether they are sufficient to support peroxisomal targeting. While most motifs were not sufficient to mediate peroxisomal localization when fused to GFP (Appendix Fig S4B), the last 10 amino acids of Yhl045w, an uncharacterized protein, were sufficient to target GFP to peroxisomes, in a Pex5 and PTS1‐binding site‐dependent manner (Fig 3C). Hence, we decided to name Yhl045w Pxp3 (Peroxisomal protein 3). The Pxp3 motif was not only sufficient but also necessary for peroxisomal targeting since a C′ fusion of a short HA tag prevented it from acting as a targeting signal (Appendix Fig S4C). Indeed, a representative frame from the MD simulations for the Pex5/Pxp3 peptide complex shows that the PTS1 of Pxp3 can nicely fit into the PTS1‐binding pocket (Appendix Fig S4D).

Experimental structures of Pex5 in complex with PTS1 cargos show that the positively charged PTS1 residue in position −2 is located in a shallow, mildly negatively charged depression within the binding cavity, but its charged end makes only water‐mediated H‐bonds with Pex5 (Stanley *et al*, 2006; Fodor *et al*, 2012). A similar shallow, mildly negative depression is seen in modeled yeast Pex5 where the tyrosine (Y) side chain in position −2 of Pxp3 can be accommodated, making numerous contacts including water‐mediated contacts that involve the OH group. To experimentally demonstrate direct binding between the potential new PTS1 and Pex5 protein and to calculate the binding affinity, we used fluorescence anisotropy, which can infer precise dissociation constants (K_d_) for the protein‐peptide interactions (Rosenthal *et al*, 2020). Indeed, this assay demonstrates that purified yeast Pex5 can directly interact with the Pxp3 targeting peptide as strongly as it binds the PTS1 of a known cargo protein, Idp3 (Fig 3D). Overall, our results confirm that Pxp3 has a *bone fide* unique PTS1, with a Y residue in position −2. This extends the consensus sequence for PTS1 motifs in yeast (Fig 3E). Interestingly, in plants, a tyrosine residue in position −2 was also shown to allow targeting of a reporter protein to peroxisomes (Lingner *et al*, 2011), supporting this finding.

Having identified a previously uncharacterized PTS1 protein, we sought to test whether the rest of the matrix proteins that rely on the Pex5 PTS1‐binding domain, but do not seem to contain a PTS1, are piggybacking on a known PTS1 protein. We designed a microscopic screen in which we recorded the peroxisomal localization of each N′ GFP‐tagged matrix protein on the background of a deletion in one known PTS1 protein. While this analysis worked well in identifying the positive controls of known piggybacking cases, we were surprised to find that no deletion of a PTS1 protein affected the localization of any newly identified peroxisomal matrix protein (data not shown), except for *Δpex8*, which obliterate the protein import of all PTS1 and PTS2 proteins as expected (Rehling *et al*, 2000). This suggests that they either piggyback on a yet unidentified peroxisomal protein or points to the possibility that these proteins directly rely on Pex5 for their peroxisomal localization without having a *bona fide* PTS1 motif.

To assess the capacity of several newly identified proteins to bind Pex5, we used a yeast‐2‐hybrid assay. We tested 10 different proteins as baits and found that only the glutathione reductase Glr1 was able to physically interact with Pex5 *in vivo* (Appendix Fig S5A). The yeast Glr1 protein was shown to be a cytosolic and mitochondrial protein (Outten & Culotta, 2004), which is important for the maintenance of the redox environment in peroxisomes (Ayer *et al*, 2012). However, the maintenance was suggested to be mediated by the cytosolic fraction of Glr1. Our whole‐proteome microscopic screen showed that when Glr1 is N′ fused to GFP, it is partially targeted to peroxisomes in a PTS1‐dependent manner (Appendix Fig S5B).

To ensure that an untagged Glr1 is indeed present and active in peroxisomes, we performed an enzymatic assay following subcellular fractionations of cells using a density gradient. We detected the peroxisomal‐ and mitochondrial‐enriched fractions by the activity of Fox2 (Fatty acid Oxidation 2) for peroxisomes and fumarase (Fum1) for mitochondria. Measuring the activity of glutathione reductase clearly demonstrates that native Glr1 is active in both peroxisomes and mitochondria (Fig 3F). These findings suggest that the yeast Glr1 is regulating the redox homeostasis of peroxisomes directly by having a small fraction targeted to peroxisomes via Pex5. How then does Glr1 bind Pex5?

Our predictions suggest that Glr1 does not have a PTS1 motif. To verify this, we (i) demonstrated that it can be targeted to peroxisomes when its most C′ is obstructed by a tag (Glr1‐mNeonGreen; Fig 3G) and (ii) measured that, unlike other PTS1 motifs, the last ten amino acids of Glr1 are not sufficient to target GFP to peroxisomes (Appendix Fig S5C). To determine which domain of Pex5 Glr1 binds, we performed another Yeast‐2‐hybrid assay, this time with one of the two structurally distinct domains of Pex5 as prey. The N′ half of Pex5 (1–299) is an intrinsically disordered domain that harbors a large number of small motifs for protein–protein interactions, while the C′ half of Pex5 (313–612) comprises the globular PTS1‐binding domain (Barros‐Barbosa *et al*, 2019). Interestingly, we found that Glr1 binds the N′ domain of Pex5 (Fig 3H).

To assess whether this interaction is direct, we expressed Pex5 and Glr1 (either individually or together) in *Escherichia coli* and tested whether they can be pulled down together *in vitro*. Our assay showed that Glr1 interacts with full‐length Pex5 and similarly to Pex5 N′ domain but only to a lesser extent with the Pex5 C′ domain (Appendix Fig S5D). Overall, our results indicate that Glr1 directly interacts with the N′ of Pex5 (1–299) and depends on the Pex5 PTS1‐binding domain for *in vivo* targeting. Previous studies have shown that the capacity of the N′ domain of Pex5 to bind the docking/translocation module on the peroxisomal membrane is “activated” when the C' domain of Pex5 binds a PTS1 protein (Barros‐Barbosa *et al*, 2019). Hence, it is possible that the capacity of the N′ domain to bind Glr1 is “activated” only when the C' binds a PTS1 protein. This could also explain why no single deletion of a PTS1 gene affected Glr1 localization. However, the single mutation in Pex5 that weakens the interaction of all PTS1 proteins (N393D) abolished Glr1 targeting (Appendix Fig S5B).

This potential dependency of the N′ Pex5 domain to bind a cargo only when the C′ Pex5 domain binds a PTS1 protein, together with the discovery of a second non‐PTS1 cargo protein that binds the N′ domain of Pex5, open a new perspective on the complexity of peroxisomal targeting and encourage a more focused endeavor to discover the mechanical properties underlying it.

### Systematic metabolomic analyses of peroxi‐ome mutants provide functional clues for newly identified peroxisomal proteins

**Figure 4:**
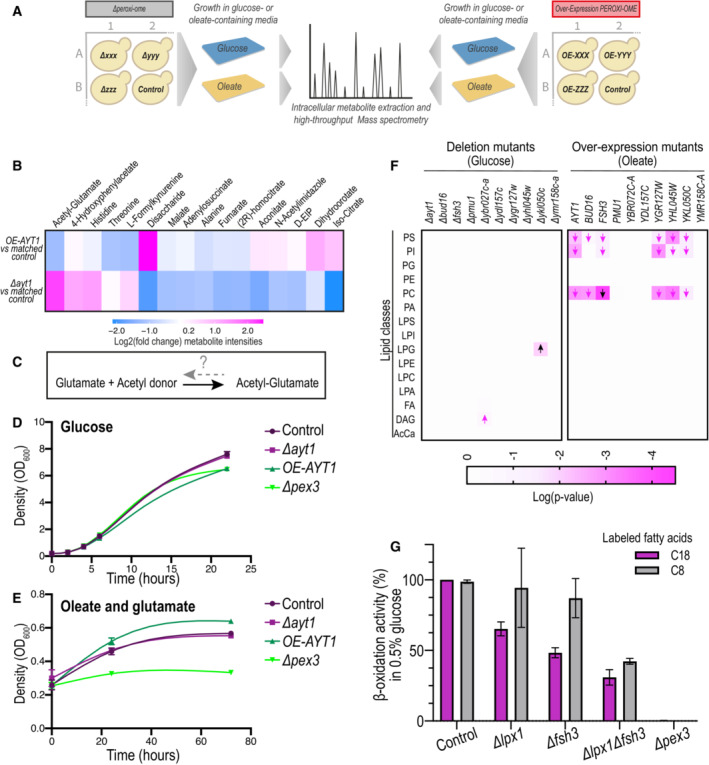
Systematic metabolomic analyses of peroxi‐ome mutants provide clues for new enzymatic functions in peroxisomes Large‐scale metabolomic analysis of peroxi‐ome mutants (either overexpression or deletion of each gene) was performed in both glucose and oleate‐containing medium to uncover uncharted metabolic functions for peroxisomal proteins in an unbiased manner. Raw data is in Datasets EV4 and EV5, circular dendrograms of all conditions are in Appendix Fig S6.Metabolomic analysis focused on strains with overexpression or deletion of the *AYT1* gene shows a significant reduction or accumulation, respectively, in acetyl‐glutamate compared to the matched control strain of each mutant. Only metabolites changing significantly in at least one of the two conditions are presented. D‐EIP is D‐erythro‐1‐(Imidazole‐4‐yl) glycerol 3‐phosphate.Acetyl‐glutamate can be generated either spontaneously in high concentrations of acetyl‐CoA and glutamate, or by Arg2 or Arg7. However, it is not known how it is catabolized.A growth assay in a condition that does not induce high levels of acetyl‐CoA in peroxisomes (glucose) shows that all strains grow similarly to the control until the stationary phase when peroxisomes become vital. In the stationary phase, the overexpression (OE) of *AYT1* grows to a lower density than the control strain, plausibly due to burdening of peroxisomal functions.A growth assay in a condition expected to elevate acetyl‐glutamate levels in peroxisomes (oleate as a sole carbon source and glutamate as a nitrogen source) demonstrates that the *AYT1* overexpressing strain grows faster and to a higher density than the control.Lipidomic analysis on mutants of 10 newly identified peroxisomal proteins whose molecular function in the yeast cell is putative or unknown shows that cells overexpressing *FSH3* had the most significant change in all conditions compared to the control strain, with a reduction of PC (raw data is in Dataset EV7). *Δykl050c* shows the most significant change in glucose conditions, with an increase of LPG lipids. Additional conditions are represented in Appendix Fig S7A. Arrows indicate the directionality of the fold‐change. Black arrows are the most significant changes in each condition. PS, phosphatidylserine; PI, phosphatidylinositol; PG, phosphatidylglycerol; PE, phosphatidylethanolamine; PC, phosphatidylcholine; PA, phosphatidic acid; “L” indicates a “lyso” phospholipid; FA, fatty acid; DAG, diacylglycerol; AcCa, acyl‐carnitine.A β‐oxidation activity assay of *Δlpx1, Δfsh3*, and *Δlpx1Δfsh3* strains supplemented with labeled 8 carbon‐ or 18 carbon‐free fatty acids in media supplemented with 0.5% glucose shows a significant reduction in β‐oxidation activity compared to the control strain and the two single mutants, suggesting an overlapping role for Fsh3 with Lpx1. This assay was done in three biologically independent replicates. Bars represent the mean and error bars represent the standard deviation. Data information: The growth assays in (D and E) were done in three biologically independent replicates and error bars were plotted. Note that some error bars are shorter than the symbol, hence are not visible in the graph.

One of our goals in expanding the peroxi‐ome was to extend our knowledge of the variety of metabolic activities carried out by peroxisomal enzymes. To realize this, we took an unbiased approach to uncover uncharted metabolic roles for peroxisomal proteins and performed a large‐scale metabolomic analysis of the peroxi‐ome. For each gene of a peroxisomal protein, we analyzed the effect of its deletion or overexpression on the yeast metabolome (thousands of chemical compounds found in the yeast cell). This experiment was performed in two growth conditions – in media containing glucose or oleate as carbon sources (Fig 4A and Datasets EV4 and EV5). This resulted in over 400 profiles of mutants and a rich source of information about the effect of these mutants on cellular metabolism.

We used hierarchical clustering to uncover the relationship between the metabolomic fingerprints of the different mutants grown on each condition and applied Gene Ontology (GO) enrichment analysis for the biological processes of known peroxisomal genes in each cluster (Appendix Fig S6A–D). This functional clustering for mutants with similar metabolome profiles can provide clues to the functions of both known and newly identified peroxisomal proteins.

When zooming into specific metabolites, we identified the previously reported activity of several newly identified peroxisomal proteins, such as a reduction in 3‐hydroxykynurenine in the deletion of the kynurenine 3‐monooxygenase, Bna4; an increase in glutathione disulfide in the absence of glutathione reductase, Glr1; and the accumulation of pyridoxine in the absence of the putative pyridoxal kinase, Bud16 (Appendix Fig S6E–G).

Confident that we can detect the changes in metabolites for known enzymes, we next focused on enzymes that contain protein domains predicted to perform specific metabolic activities, although their exact enzymatic activity was not yet defined. One such protein is Ayt1 (Acetyltransferase 1), which was predicted to be an acetyltransferase by sequence similarity to the *Fusarium sporotrichioides* acetyltransferase, Tri101 (Alexander *et al*, 2002), but whose molecular function in baker's yeast was never studied. Further metabolomics focused on the deletion and overexpression of *AYT1* revealed a significant change in the levels of acetyl‐glutamate compared to the equivalent control strain (Fig 4B and Dataset EV6). This observation is interesting because the acetyltransferase domain could recognize acetyl‐glutamate.

In yeast, acetyl‐glutamate is generated by two mitochondrial enzymes, Arg2 and Arg7, and can also form non‐enzymatically in conditions of high concentrations of acetyl‐CoA and glutamate (Dercksen *et al*, 2016) such as those found in peroxisomes during growth on oleate. However, how acetyl‐glutamate is catabolized in yeast is not known. We hypothesized that Ayt1 may be important to regenerate glutamate from acetyl‐glutamate by either using an acetyltransferase or esterase activity (Fig 4C). To test this hypothesis in a physiological context, we performed a growth assay for strains with either a deletion or overexpression of *AYT1* in a condition that can elevate the amount of intra‐peroxisomal acetyl‐glutamate. This medium contains glutamate as the nitrogen source, in combination with oleate as the sole carbon source, that, upon β‐oxidation, generates acetyl donors in peroxisomes. Acetyl‐CoA can react with glutamate and increase acetyl‐glutamate levels (Dercksen *et al*, 2016). Interestingly, while all strains grew similarly to the control during the logarithmic phase in glucose conditions, the overexpression (OE) of *AYT1* and *Δpex3* strains grew slower than the control during the stationary phase (22 h into the growth assay; Fig 4D). We hypothesize that during the stationary growth phase, when glucose is deprived and peroxisomes become important for viability, the overexpression of a peroxisomal protein might add a burden on their function or even compete with their targeting machinery. Importantly, however, in the oleate and glutamate condition, the *OE‐AYT1* strain grew faster and to a higher density than the control (Fig 4E). We speculate that during oleate growth, when β‐oxidation generates large amounts of reactive acetyl‐CoA units in the peroxisomal matrix, Ayt1 becomes beneficial for regenerating glutamate. In these conditions, since the targeting machinery is upregulated, the overexpression of Ayt1 may not be a burden. This result is in line with the metabolomic analysis, which altogether suggests that Ayt1 catabolizes acetyl‐glutamate and facilitates growth under oleate conditions.

Since our metabolomic analysis exposed a new potential enzymatic role for Ayt1, we wanted to examine the involvement of several newly identified peroxisomal proteins in lipid metabolism, as one of the hallmarks for peroxisome function is the degradation of fatty acids. We, therefore, performed lipidomic analysis for strains with either a deletion or an overexpression in one of the 10 newly identified peroxisomal proteins whose molecular function in yeast was unknown or putative according to the *Saccharomyces* Genome Database (SGD; Cherry *et al*, 2012). To get a clearer view of the overall changes in the lipidomic profile of the strains, we grouped different lipids according to their classes (Dataset EV7, Fig 4F, and Appendix Fig S7A). Intriguingly, the most significant change in all conditions was observed for the overexpression of Fsh3 in oleate‐growing cells, causing a reduction in phosphatidylcholine (PC; Fig 4F). Fsh3 is a putative lipase that belongs to the family of serine hydrolases (Baxter *et al*, 2004). Fsh3 was predicted by sequence to contain a PTS1 motif (Notzel *et al*, 2016), a prediction supported by our MD computations (Appendix Fig S4A). Moreover, in a functional proteome assay in which serine lipid hydrolases were pulled down by labeled inhibitors, Fsh3 was detected in the peroxisomal fraction (Ploier *et al*, 2013). We confirmed that Fsh3, which contains a unique PTS1 sequence (G in position −3), is indeed a matrix protein (Fig 2E), and that its C′ motif functions as a PTS1 – its last 10 amino acids mediate GFP targeting to peroxisomes in a Pex5‐dependent manner (Appendix Fig S7B).

To check whether Fsh3 has an overlapping function with the only peroxisomal lipase characterized to date, Lpx1 (Lipase of Peroxisomes 1; Thoms *et al*, 2008), we measured the β‐oxidation activity of cells lacking either one of the two enzymes, *Δlpx1* and *Δfsh3*, or both enzymes *Δlpx1Δfsh3*. As expected, when cells grew in oleate, none of the deletions affected β‐oxidation activity (Appendix Fig S7C), plausibly due to high levels of free fatty acids in the media, which would not require lipase activity. However, in low (0.5%) glucose conditions, cells first store lipids as tri‐acylglycerols before sugar became sparse and only then would breakdown of the phospholipids require a lipase activity. In these conditions, the double mutant *Δlpx1Δfsh3* showed a significant reduction in β‐oxidation activity compared to the control strain and the two single mutants *Δlpx1* and *Δfsh3* (Fig 4G). This effect was observed regardless of the labeled fatty acid that was added, the medium‐chain fatty acid C8 or the long‐chain fatty acid C18. Our findings alongside studies showing Fsh3 is a putative lipase (Baxter *et al*, 2004; Ploier *et al*, 2013), suggest that Fsh3 is a newly identified PTS1‐containing peroxisomal lipase.

A deeper inspection of previous studies on putative lipases (Ploier *et al*, 2013) brought to our attention that Ykl050c possesses a clear lipase activity; however, its cellular localization was never clearly determined. Our work uncovers also Ykl050c as part of the peroxi‐ome and the lipidomic results show that *Δykl050c* had the most significant effect on the yeast lipidome when cells grew in glucose‐containing media. This strain showed significantly higher levels of lyso‐phosphatidylglycerol (LPG; Fig 4F). The identification of Ykl050c as a peroxisomal matrix protein (Fig 2D) alongside our lipidomic results and its clear lipase activity as previously reported (Ploier *et al*, 2013) propose that Ykl050c is an additional newly identified peroxisomal lipase, hence we named Ykl050c, Lpx2 (Lipase of Peroxisomes 2).

In summary, we propose that the peroxisomal matrix may contain three peroxisomal lipases (Lpx1, Lpx2, and Fsh3) that potentially act on different lipid substrates. Overall, our systematic approach revealed the potential activity of three new metabolic enzymes in peroxisomes, Ayt1, Fsh3, and Lpx2.

### Uncovering peroxisomal targeting of GID complex subunits places peroxisomes as regulators of gluconeogenesis

**Figure 5:**
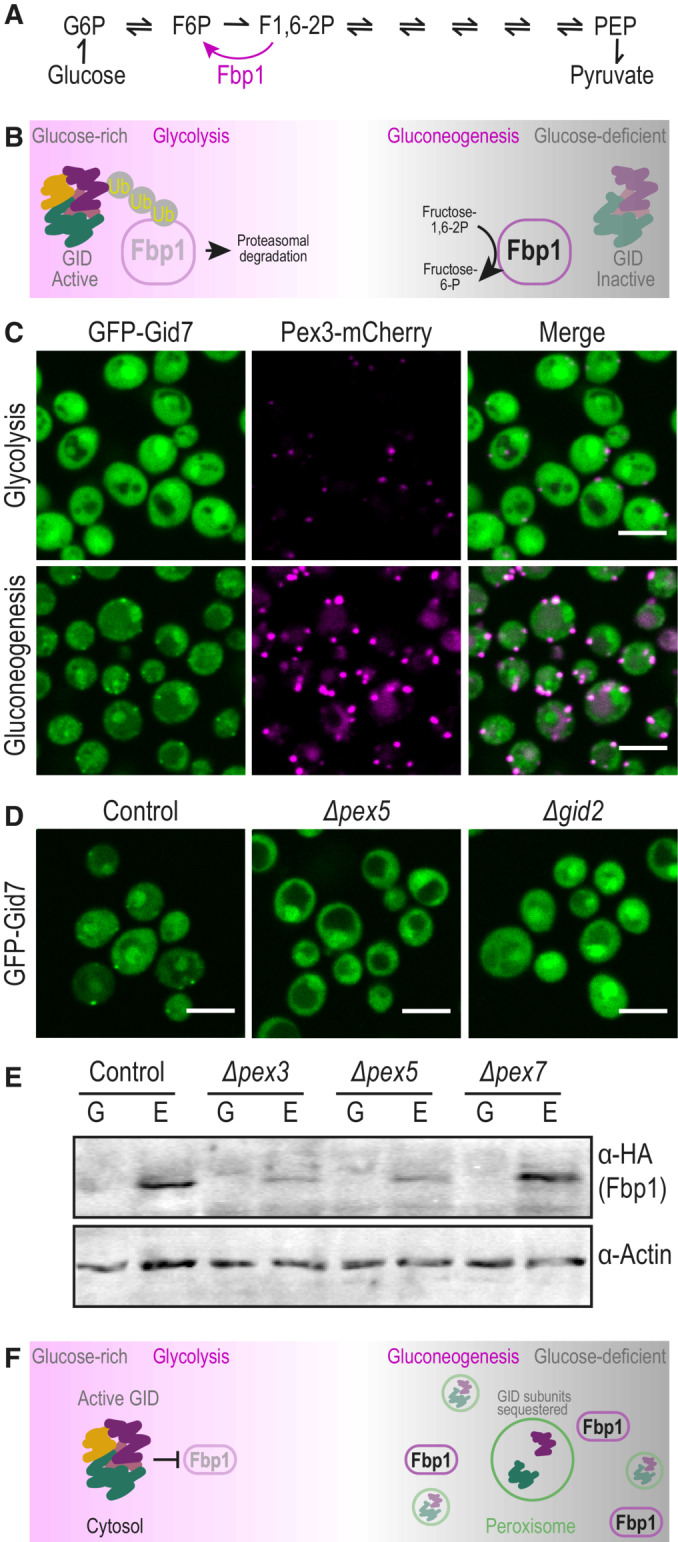
Uncovering peroxisomal targeting of GID complex subunits places peroxisomes as regulators of gluconeogenesis Gluconeogenesis utilizes unique enzymes that circumvent irreversible steps of glycolysis, one of them is the Fructose‐1,6‐bisphosphatase Fbp1.To ensure that gluconeogenesis does not work during glucose replete conditions, the GID complex ubiquitinates gluconeogenic enzymes shortly after glucose addition to gluconeogenic cells and marks them for proteasomal degradation. In gluconeogenic conditions, the subunit that provides substrate selectivity, Gid4, is eliminated, enabling the upregulation of necessary gluconeogenic enzymes.GFP‐Gid7, a subunit of unknown function of the GID complex, is targeted to peroxisomes in gluconeogenic conditions. The images in this panel are identical to Appendix Fig S8A Glucose (logarithmic) and Glucose (stationary) images. GFP‐Gid2, which was previously shown to co‐localize with peroxisomes, shows a similar phenotype (Appendix Fig S8B).The peroxisomal localization of GFP‐Gid7 is dependent on both *PEX5* and *GID2*, implying that the targeting of Gid7 is mediated in the context of the complex or a sub‐complex.Western blot analysis of Fbp1‐HA levels in mutants *Δpex3* (no peroxisomes), *Δpex5* (abolished targeting of GID subunits to peroxisomes), and *Δpex7* (no β‐oxidation due to loss of Pot1 targeting) demonstrates that 2 h after the transition from glucose to ethanol (gluconeogenic conditions), control and *Δpex7* cells upregulated Fbp1‐HA levels, while for *Δpex3* and *Δpex5* cells Fbp1‐HA levels remain low. Antibodies were used against the HA tag (for Fbp1) and Actin as a loading control.Peroxisomes regulate Fbp1 levels in the transition to gluconeogenic conditions, plausibly due to partial sequestration of GID complex subunits in the peroxisomal matrix. Data information: For all micrographs, a single focal plane is shown. The scale bar is 5 μm.

The establishment of a comprehensive compendium of peroxisomal proteins in an unbiased and non‐hypothesis‐driven manner brought to light peroxisomal localization of several well‐known proteins that would not have been suspected to be in peroxisomes. We were particularly intrigued by the co‐localization of two Glucose Induced degradation Deficient (GID) complex subunits with peroxisomes when cells are grown in oleate‐containing media (Dataset EV1). The GID complex is a well‐known and highly studied ubiquitin ligase that regulates glucose homeostasis, a fundamental cellular process tightly controlled by two opposing metabolic pathways – the breakdown of glucose through glycolysis and the regeneration of glucose by gluconeogenesis. These two metabolic processes share most enzymes; however, few steps are irreversible (Melkonian *et al*, 2020). Gluconeogenesis utilizes unique enzymes that circumvent irreversible steps of glycolysis, one of them is mediated by the enzyme fructose‐1,6‐bisphosphatase (Fbp1; Fig 5A).

The GID complex is active during the transition to glucose‐rich conditions when it polyubiquitinates gluconeogenic enzymes and marks them for degradation by the proteasome (Fig 5B; Regelmann *et al*, 2003; Menssen *et al*, 2012). Shortly after addition of glucose to gluconeogenic cells, Gid4 (also called Vid24), the subunit that provides substrate binding and selectivity, is upregulated and afterward eliminated (Menssen *et al*, 2018). Hence, it is not surprising that we detect both GFP‐Gid7 and GFP‐Gid2 mainly localized to the cytosol in glucose‐rich conditions (Fig 5C). Interestingly, however, GFP‐Gid2 and GFP‐Gid7 localized mainly to peroxisomes in gluconeogenic conditions, i.e., media with non‐fermentable carbon sources like oleate, ethanol, and glycerol, or growth during stationary phase, when glucose is depleted (Fig 5C, and Appendix Fig S8A and B) (the images in Fig 5C are identical to Appendix Fig S8A Glucose (logarithmic) and Glucose (stationary) panels). Importantly, some weak cytosolic signal was detected for GFP‐Gid2 and GFP‐Gid7; however, these proteins were expressed under a constitutive promoter (*NOP1pr*), hence, it could be that the weak cytosolic signal is a result of an expression that is higher than their native level.

Moreover, our sub‐organelle localization analysis showed that Gid2 and Gid7 were localized in the peroxisomal matrix in gluconeogenic conditions (Appendix Fig S8C) and our targeting analysis showed that they were dependent on Pex5 (Dataset EV1). Although Gid7 and Gid2 do not interact directly in the complex (Menssen *et al*, 2012) we found that GFP‐Gid7 was also dependent on Gid2 for proper targeting into peroxisomes (Fig 5D), implying that the targeting of Gid7 is mediated in the context of the complex or a sub‐complex. However, we could not observe other subunits inside peroxisomes using tagging and fluorescence microscopy.

What would GID subunits be doing in peroxisomes only in gluconeogenic conditions, when the complex is transitioning to an inactive state? We first assayed if GID subunits had a role inside peroxisomes but found no major effects for *∆gid2* or *∆gid7* on the levels and localization of peroxisomal matrix proteins using fluorescence microscopy (data not shown). We hence hypothesized that the possible role of peroxisomes is to sequester the subunits away from the cytosol to form an additional layer of regulation in shutting down the function of this complex during gluconeogenesis. To test this hypothesis, we created strains mutated in peroxisome biogenesis and checked the levels of Fbp1 in the transition from glucose to ethanol. We observed that 2 h after the transition to ethanol, the control cells already managed to upregulate the levels of Fbp1‐HA. However, in *Δpex3* cells that lack peroxisomes (Höhfeld *et al*, 1991) and hence have GID complex members still cytosolic, and in *Δpex5* cells in which the GID subunits are not targeted to peroxisomes, Fbp1‐HA levels remained low. This effect was specific and not a by‐product of reduced fatty acid catabolism since the deletion of *PEX7*, the peroxisomal targeting factor that is not mediating the targeting of GID subunits (Dataset EV1), did not affect Fbp1 levels (Fig 5E). This demonstrates that fully formed peroxisomes, as well as Pex5‐dependent targeting, are regulating Fbp1 levels in the transition to gluconeogenesis, plausibly due to partial sequestration of GID complex subunits in the peroxisomal matrix (Fig 5F).

## Discussion

Defining the extent of the peroxi‐ome and its multifaceted functionality has been a focus and a challenge of the field for the past 70 years. Indeed, tens of proteins were identified so far in yeast peroxisomes and many of them were functionally studied in great detail (Chen & Williams, 2018). Building on the assumption that more remains to be uncovered, we used a high‐content screening approach and expanded the protein count of peroxisomes by ~40%. This comprehensive inventory of peroxisomal proteins provides a broad basis for the systematic investigation of peroxisomes, allowing an in‐depth understanding of how defects in peroxisomes lead to diseases. Indeed, over 60% of the yeast peroxi‐ome have an established human homolog (Dataset EV1; Cherry *et al*, 2012).

Why were the newly uncovered peroxisomal proteins never assigned to peroxisomes in the past? First, previous proteomic approaches relied on the enrichment of proteins in certain fractions reducing the chances of clearly classifying dual localized proteins correctly. In addition, many of the newly described peroxisomal proteins are expressed at low levels in the conditions that were previously tested. This, in combination with the fact that most of the proteins we found do not contain a canonical targeting sequence, and hence could not have been detected by sequence analysis, exemplifies the sensitivity and novelty of our systematic imaging approach.

We believe that more peroxisomal proteins may be identified using imaging approaches. For example, proteins whose N′ is important for their peroxisomal targeting, must in the future be examined by fusing their C′ to a fluorophore. Moreover, some proteins are localized to peroxisomes only in specific conditions even when expressed under a constitutive *NOP1pr*, hence visualizing them under different growth conditions could expose their peroxisomal localization. Still, having this first comprehensive list enables us to leverage our extensive peroxi‐ome view to perform systematic analyses never before possible on an organelle‐wide level. These, alongside dedicated follow‐up experiments, have already enabled the discovery of several, fundamental, concepts in peroxisome biology ranging from targeting, through metabolic, to regulatory mechanisms.

First, we uncovered a striking phenomenon whereby all 22 newly identified matrix proteins relied not only on Pex5 but specifically on the PTS1‐binding activity of Pex5, although most of them did not include a PTS1 motif. One possibility is that only a small fraction of each of these proteins interact with Pex5 by having a cryptic PTS1 motif, which is not represented in the gene's sequence. For example, in mammalian cells, it was shown that a translational readthrough can result in the creation of a PTS1 motif for a small fraction (1.8%) of the Lactate Dehydrogenase B (LDHB) protein (Schueren *et al*, 2014) and a small fraction (4%) of Malate Dehydrogenase 1 (MDH1) protein (Hofhuis *et al*, 2016). Other mechanisms to expose a cryptic PTS1 motif include an alternative splicing (Freitag *et al*, 2012) and translational frameshift (Malagnac *et al*, 2013). Hypothetically, any type of manipulation – from cleavage to post‐translational modification can result in a modified C′, which may lead to a creation of a PTS1‐like motif. Having now exposed tens of cases of non‐PTS1 Pex5‐dependent proteins enables the investigation of, what seems to be, significant alternative mechanisms to target proteins into peroxisomes.

Another concept that came to light by our functional analysis is the identification of two additional potential lipases in peroxisomes, Ykl050c (Lpx2) and Fsh3. Why would peroxisomes need to house a lipase, not to mention three of them, if the current dogma for lipid metabolism in peroxisomes involves the transport of free fatty acids or CoA conjugated fatty acids (acyl‐CoAs) for β‐oxidation (van Roermund *et al*, 2021)? A fascinating recent observation from *Arabidopsis thaliana* peroxisomes showed that peroxisomes form intraluminal vesicles with roles in fatty acid catabolism and protein compartmentalization (Wright & Bartel, 2020). In yeast, electron microscopy images of *Δlpx1* peroxisomes showed abnormal morphology with intraperoxisomal vesicles (Thoms *et al*, 2008). In the vacuole, the lipase Atg15 is known to degrade lipid vesicles (Epple *et al*, 2001; Teter *et al*, 2001), suggesting that peroxisomal lipases may have a similar role in degrading such intraluminal vesicles as a source for free fatty acids and/or for membrane remodeling. Indeed, our high‐resolution imaging demonstrated that Fsh3 compartmentalizes in the matrix to a specific niche. Moreover, deletions of both *Δlpx1* and *Δfsh3* reduce β‐oxidation activity specifically when cells are grown in low glucose when there is no excess of free fatty acids in the cell. All of the above may suggest that the peroxisomal lipases Lpx1, Lpx2, and possibly also Fsh3, act on the peroxisomal membrane or on intraperoxisomal vesicles to release fatty acids for β‐oxidation and in doing so also help to maintain normal peroxisomal membrane morphology. These findings suggest that lipid catabolism in peroxisomes is more complex than is currently thought and urge further research on the function of peroxisomal lipases.

Finally, the identification of peroxisomal targeting of GID complex subunits in gluconeogenic growth conditions adds a mechanistic view to the important concept of peroxisomes as carbon‐source regulatory organelles. It has been well appreciated that peroxisomes are important for gluconeogenesis as they regenerate acetyl‐CoA during β‐oxidation. Subsequently, the acetyl‐CoA can be fed into the glyoxylate cycle and the citric acid cycle to produce metabolites for gluconeogenesis (Masters, 1997; Jardón *et al*, 2008). However, our results propose a regulatory role that goes beyond the simple provision of building blocks. In the same conditions that peroxisomes are most metabolically active, and in which Pex5 is upregulated, they also ensure the parallel essential presence of gluconeogenesis, plausibly by sequestering GID complex subunits and enabling the stabilization of a central enzyme in the pathway Fbp1.

The GID complex has been highly conserved through evolution in terms of sequence; however, in terms of functionality, it evolved a change in substrates. The human GID complex still regulates metabolism (Leal‐Esteban *et al*, 2018); however, the gluconeogenic enzymes FBP1 and Phosphoenolpyruvate Carboxy‐Kinase (PCK1) are not direct targets of the GID complex (Lampert *et al*, 2018). Despite that, its co‐evolution with peroxisomes seems to have persisted. For example, the murine GID complex was found to ubiquitinate AMP‐activated kinase (AMPK) and therefore negatively regulate its function (Liu *et al*, 2020). AMPK is a regulator of cellular energy homeostasis – once activated it inhibits the transcription of gluconeogenic enzymes in the liver (Lochhead *et al*, 2000). In mouse hepatocytes, a deletion of PEX5 perturbed gluconeogenesis through an unknown mechanism that did, however, involve AMPK activation (Peeters *et al*, 2011). It remains to further investigate whether peroxisomes regulate GID complex function also in higher eukaryotes and whether the regulation of gluconeogenesis occurs through AMPK degradation.

Interestingly, the mammalian GID complex was shown to target the transcription factor HBP1 for degradation, and thereby regulates cell proliferation (Lampert *et al*, 2018). Among our list of newly found peroxisomal proteins, several proteins are known to be involved in cell cycle and genome duplication: the spindle pole body component Nud1 and its interactor Ady3; cell cycle regulators Mps1 and Tyc1; cell division regulator Afr1; the histone H3‐like protein required for kinetochore function Cse4; and a component of the synaptonemal complex (involved in meiotic crossing over), Gmc2. Although all proteins were expressed under a constitutive promoter, Tyc1 co‐localized with peroxisomes more robustly in glucose conditions compared to oleate. Tyc1 is an inhibitor of the Anaphase‐Promoting Complex/Cyclosome (APC/C), a ubiquitin ligase that promotes different cell cycle phases (Schuyler *et al*, 2018). Yeast cells proliferate more rapidly when grown on glucose compared to non‐fermentable carbon sources like oleate. Intriguingly, even when peroxisomes are artificially induced in glucose‐containing media by the expression of engineered transcription factors important for peroxisome proliferation, this results in a growth delay (Grewal *et al*, 2021), implying that peroxisome function and the cell cycle are co‐regulated. An interesting hypothesis to test in the future is whether Tyc1 is sequestered in peroxisomes to allow the proper function of APC/C in glucose‐containing media. This also raises the much more global question regarding the role of peroxisomes and their activity in affecting cell cycle progression in response to metabolic changes. More globally our findings on the GID complex sequestration and the hypothesis on APC/C regulation support the idea that peroxisomes can be used to rapidly sequester regulatory proteins away from the cytosol (Reglinski *et al*, 2015).

In conclusion, our findings highlight that current knowledge on peroxisomes is only the tip of the iceberg. The discovery of multiple peroxisomal proteins introduces a more holistic perception of the peroxi‐ome and the various enzymatic and regulatory activities that it may hold. More broadly, with the new understanding of the importance of peroxisomes in cellular and organismal physiology (Zalckvar & Schuldiner, 2022), we provide important insights that highlight new links between peroxisome regulatory and enzymatic function to carbon‐source dependent cellular behavior.

## Materials and Methods

### Yeast strains and strain construction

All strains in this study are based on the BY4741 laboratory strain (Brachmann *et al*, 1998), except for strains based on CB199 (see the complete list of yeast strains and primers in Dataset EV8). The libraries used were: (i) the yeast SWAT N′‐GFP library, which is a collection of 5,457 strains tagged with GFP at their N′ and expressed under a generic, constitutive, promoter (SpNOP1pr; Weill *et al*, 2018). 1/3 of the library was examined previously (Yifrach *et al*, 2016), and the additional 2/3 of the library was examined in this study, (ii) the yeast peroxisomal mini deletion library, and (iii) the yeast overexpression (*TEF2pr*‐mCherry) library. Cells were genetically manipulated using a transformation method that includes the usage of lithium‐acetate, polyethylene glycol, and single‐stranded DNA (Daniel Gietz & Woods, 2002). Plasmids are described in Dataset EV9. The pYM‐based pMS555 plasmid that was originally used for the N‐terminal GFP tagging (Yofe *et al*, 2016; Weill *et al*, 2018) was modified to contain the last 10 aa of Pxp3 (Fig 3C), Gid7, Nud1, Ybr072c‐a (Appendix Fig S4B) and Fsh3 (Appendix Fig S7B) at the C′ of the GFP sequence. These constructs were genomically integrated into the HO locus in strains containing Pex3‐mCherry, with or without *pex5* deletion or *PEX5* N393D point mutation. Primers for validation of correct locus insertion were designed using the Primers‐4‐Yeast website (Yofe & Schuldiner, 2014).

### Yeast growth media

Synthetic media used in this study contains 6.7 g/l yeast nitrogen base with ammonium sulfate (Conda Pronadisa #1545) and either 2% glucose, 2% ethanol, 3% glycerol, or 0.2% oleic acid (Sigma) +0.1% Tween 80, with complete amino acid mix (oMM composition, Hanscho *et al*, 2012), unless written otherwise; when Hygromycin or Geneticin antibiotics were used, media contains 0.17 g/l yeast nitrogen base without Ammonium Sulfate (Conda Pronadisa #1553) and 1 g/l of monosodium glutamic acid (Sigma‐Aldrich #G1626) instead of yeast nitrogen base with ammonium sulfate. When mentioned, 500 mg/l Hygromycin B (Formedium), 500 mg/l Geneticin (G418; Formedium), and 200 mg/l Nourseothricin (WERNER BioAgents “ClonNat”) were used.

### Yeast library preparation

To create collections of haploid strains containing GFP‐tagged proteins with additional genomic modification such as a peroxisomal marker (Pex3‐mCherry and Pex11‐mScarlet) or different deletions *(Δpex5* and *Δpex7*) and point mutations (*PEX5 Y253N* and *PEX5 N393D*), different query strains were constructed based on an SGA compatible strain (for further information see Dataset EV8). Using the SGA method (Tong & Boone, 2006; Cohen & Schuldiner, 2011) the Pex3‐mCherry query strain was crossed with 2/3 of the SWAT N′‐GFP library and the other query strains were crossed into a collection of strains from the SWAT N′‐GFP library containing ~90 strains including known and newly identified peroxisomal proteins together with controls. To perform the SGA in a high‐density format we used a RoToR benchtop colony arrayer (Singer Instruments). In short: mating was performed on rich medium plates, and selection for diploid cells was performed on SD‐URA plates containing query strain‐specific antibiotics. Sporulation was induced by transferring cells to nitrogen starvation media plates for 7 days. Haploid cells containing the desired mutations were selected by transferring cells to SD‐URA plates containing the same antibiotics as for selecting diploid cells, alongside the toxic amino acid derivatives 50 mg/l Canavanine (Sigma‐Aldrich) and 50 mg/l Thialysine (Sigma‐Aldrich) to select against remaining diploids, and lacking Histidine to select for spores with an A mating type. To create the peroxisomal mini deletion library, the BY4741 laboratory strain was transformed using plasmid pMS047 (Dataset EV9) and with primers designed by the Primers‐4‐Yeast website for each peroxisomal gene.

### Automated high‐throughput fluorescence microscopy

The collections were visualized using an automated microscopy setup as described previously (Breker *et al*, 2013). In short: cells were transferred from agar plates into 384‐well polystyrene plates for growth in liquid media using the RoToR arrayer robot. Liquid cultures were grown in a LiCONiC incubator, overnight at 30°C in an SD‐URA medium. A JANUS liquid handler (PerkinElmer) connected to the incubator was used to dilute the strains to an OD_600_ of ~0.2 into plates containing SD medium (6.7 g/l yeast nitrogen base and 2% glucose) or S‐oleate (6.7 g/l yeast nitrogen base, 0.2% oleic acid and 0.1% Tween‐80) supplemented with –URA amino acids. Plates were incubated at 30°C for 4 h in SD medium or for 20 h in S‐oleate. The cultures in the plates were then transferred by the liquid handler into glass‐bottom 384‐well microscope plates (Matrical Bioscience) coated with Concanavalin A (Sigma‐Aldrich). After 20 min, wells were washed twice with SD‐Riboflavin complete medium (for screens in glucose) or with double‐distilled water (for screens in oleate) to remove non‐adherent cells and to obtain a cell monolayer. The plates were then transferred to the ScanR automated inverted fluorescence microscope system (Olympus) using a robotic swap arm (Hamilton). Images of cells in the 384‐well plates were recorded in the same liquid as the washing step at 24°C using a 60× air lens (NA 0.9) and with an ORCA‐ER charge‐coupled device camera (Hamamatsu). Images were acquired in two channels: GFP (excitation filter 490/20 nm, emission filter 535/50 nm) and mCherry (excitation filter 572/35 nm, emission filter 632/60 nm).

### Manual microscopy

Manual microscopy imaging was performed with the following strains: GFP‐Nud1 with Pex3‐mCherry or Spc42‐mCherry (Fig 1C); GFP‐Last 10 aa of Pxp3 (Fig 3C), Gid7, Nud1, Ybr072c‐a (Appendix Fig S4B) and Fsh3 (Appendix Fig S7B); GFP‐Pxp3 control GFP‐Pxp3‐HA (Appendix Fig S4C); Glr1‐mNeonGreen (Fig 3G); GFP‐Gid7 in different growth conditions (Fig 5C and Appendix S8A and B) and with genetic manipulations (Fig 5D). Yeast strains were grown as described above for the high‐throughput microscopy with changes in the selection required for each strain (See yeast strain information in Dataset EV8). Imaging was performed using the VisiScope Confocal Cell Explorer system, composed of a Zeiss Yokogawa spinning disk scanning unit (CSU‐W1) coupled with an inverted Olympus microscope (IX83; ×60 oil objective; Excitation wavelength of 488 nm for GFP). Images were taken by a connected PCO‐Edge sCMOS camera controlled by VisView software.

### High‐resolution imaging

The collection of yeast strains with N′‐GFP tagged peroxi‐ome proteins and Pex11‐mScarlet were transferred manually from agar plates into 384‐well polystyrene plates (Greiner) for growth in SD‐URA liquid media. Liquid cultures were grown in a shaking incubator (Liconic), overnight at 30°C. Then, strains were diluted to an OD_600_ of ~0.2 into plates with S‐oleate media. Strains were incubated for 20 h at 30°C to induce enlargement of peroxisomes and transferred manually into glass‐bottom 384‐well microscope plates (Matrical Bioscience) coated with Concanavalin A (Sigma‐Aldrich). After 20 min, cells were washed three times with double‐distilled water to remove non‐adherent cells and to obtain a cell monolayer. The plate was then imaged in an automated inverted fluorescence microscope system (Olympus) harboring a spinning disk high‐resolution module (Yokogawa CSU‐W1 SoRa confocal scanner with double microlenses and 50 μm pinholes). Images of cells in the 384‐well plates were recorded in the same liquid as the washing step at 30°C using a 60× oil lens (NA 1.42) and with a Hamamatsu ORCA‐Flash 4.0 camera. Fluorophores were excited by a laser and images were recorded in two channels: GFP (excitation wavelength 488 nm, emission filter 525/50 nm) and mScarlet (excitation wavelength 561 nm, emission filter 617/73 nm). All images were taken in a Z‐stack and using cellSens software. The best focal plane showing the “ring‐like” structure of the peroxisomal membrane was chosen for defining sub‐organellar localization. For presentation, images were deconvoluted using cellSens software.

### Molecular dynamics (MD) simulations

Exploring the association of a peptide with the Pex5 receptor via MD simulations would require excessively long computations. We, therefore, chose to explore the stability of Pex5‐peptide complexes. Each simulation started from a Pex5‐peptide complex modeled based on the experimental structure of human Pex5‐cargo complexes, and it was assumed that improbable or unstable complexes would dissociate or weaken as the simulation proceeds. Two 100 ns trajectories were calculated for each complex. The last 90 ns of the two trajectories were analyzed together, providing estimates of selected contacts stability for a combined 180 ns simulation. To estimate the effect of time on the Pex5‐peptide contacts, the analyses were repeated for the last 50 ns of the two trajectories, 100 ns altogether.

Our analyses focused on the direct hydrogen (H)‐bond interactions between the backbone of the peptide and all polar/charged side chains lining the PTS1 binding cavity of Pex5, defining an H‐bond as a distance of 0.35 nm or less between H‐bond donors and acceptors. The analysis distinguishes between backbone oxygen atoms, which can each accept two or more H‐bonds, and backbone nitrogen atoms that can each donate one H‐bond. The C′ Ot atoms can accept four H‐bonds and these are individually analyzed, and so are the two H‐bonds that O‐2 accepts. Notably, distance analyses showed that the Nε and Nη atoms of Pex5 Arg526 are often at an H‐bond distance from peptide atom O‐2. However, the direction of Arg526 N‐H bonds is inadequate for H‐bond formation therefore these contacts were not considered as H‐bonds in the analyses. O‐3 and O‐5 form only one H‐bond contact in the experimental structures but in the MD trajectories H‐bonds were also formed to the sidechain of Tyr468. This residue replaces N462 in human Pex5 and being larger, it protrudes into the PTS1 binding cavity. The details of individual contacts are given in Dataset EV2 but the analyses considered the sum of the two contacts for each backbone oxygen. For backbone N‐3 and N‐5 atoms, the analysis considers only the shortest H‐bond distance in each MD frame.

MD simulations for human Pex5 in complex with peptide YQSKL (entry 1FCH in the Protein Data Bank) were used as a test case. Most of the H‐bond contacts seen in the experimental structure are well preserved in the MD trajectories, as detailed in Dataset EV2. The Ot atoms make stable H‐bonds with Asn378/Nδ2 (85.5% of the trajectory) and Asn489/Nδ2 (62.0% of the trajectory), as seen in the experimental structure. They also form very frequent H‐bonds with the sidechains of Lys490 and Arg520, 43.6 and 93.6% of the trajectory, respectively, which in the experimental structures make only water‐mediated H‐bonds with the cargo. Most H‐bonds of peptide residues −2 and −3 are also well preserved in the human Pex5 complex simulation (> 50% of the time); less preserved is the contact N‐2 with Asn524/Oδ1, maintained only 36.1% of the time. In contrast, the H‐bonds formed by the backbone of residue −5 are not stable in the simulations. This result is not surprising as in the experimental structure residue Y‐5 is also stabilized through contacts with a neighboring molecule related by crystal symmetry, while the MD simulation is for a single molecular complex.

Hydrogen bond stabilities throughout the MD trajectories for 22 known yeast Pex5 cargos were used to establish a plausible measure for identifying stable binders among the new peroxisomal proteins. The most stable hydrogen bonds were formed by the backbone atoms of peptide residues −1 and −3. The ranges of the averaged hydrogen‐bond stabilities for each of these residues distinguished well between the known cargos and Mdh2, a known non‐cargo, and thus were used to identify stable binders (Dataset EV3). The time dependence of the H‐bonds stability is minor, indicating that rapid structural changes occur in the first 10 ns of the trajectory, which are not included in the analysis.

MD simulations were executed with the Gromacs package (Van Der Spoel *et al*, 2005). Trajectory analyses were performed with Gromacs and with programs written by M.E. UCSF‐chimera (Pettersen *et al*, 2004) was used to model starting structures of the Pex5/peptide complexes and to produce Appendix Fig S4D.

### Pex5 protein purification for fluorescence anisotropy

Full‐length *Saccharomyces cerevisiae* Pex5 was cloned in a petM30 vector. Pex5 was expressed in autoinduction medium (Studier, 2005), with 5 h at 37°C and 26 h at 20°C. Cells were harvested, resuspended in lysis buffer (50 mM Hepes pH 7.5, 150 mM NaCl, 20 mM imidazole, protease inhibitor (Roche), DNAse (Sigma), and lysozyme (Sigma)), homogenized 1 h at 4°C and lysed by sonication. The lysate was then cleared by centrifugation and the supernatant was loaded onto Ni‐NTA resin. Bound proteins were washed with 50 mM Hepes pH 7.5, 750 mM NaCl, 20 mM Imidazole, and the protein was eluted with 50 mM Hepes pH 7.5, 150 mM NaCl, 250 mM Imidazole. The eluate was then dialyzed against Hepes pH 7.5, 150 mM NaCl, 0.5 mM TCEP and simultaneously digested with 1 mg of TEV‐protease. Undigested protein and TEV protease were removed by a second Ni‐NTA step and flow through containing Pex5p was concentrated for gel filtration (Hiload 16/60 Superdex 200 pg, GE healthcare). Relevant fractions were pooled and the protein was concentrated, flash‐frozen in liquid nitrogen, and stored at −80°C.

### Fluorescence anisotropy

Fluorescein isothiocyanate (FITC) labeled peptides corresponding to the carboxyl‐terminal 10 amino acids of Yhl045w (FITC‐RKRVLGVAYL, Genscript) and Idp3 (FITC‐YEDKKGMCKL, Genscript) were solubilized in water and used in the assay at a final concentration of 10 nM. A tyrosine was added at the N terminal of Idp3 for concentration determination. Measurements of fluorescence anisotropy changes were performed in black 96‐well plates (Greiner) with an Infinite M1000 plate reader (TECAN) with excitation/detection at 470/530 nm. The experiment was performed in 50 mM Hepes pH 7.5, 150 mM NaCl. A concentration range from 38 to 120 nM (for Idp3) or 20 to 150 nM (for Yhl045w) was obtained by serial dilution and each concentration was measured in triplicate. Three independent experiments were performed and binding data were normalized and analyzed using Prism (GraphPad software, USA). Kinetic information was obtained by least‐square fitting of a binding–saturation model with one binding site.

### Yeast‐2‐hybrid assay for in‐vivo interactions with Pex5

PJ69‐4A cells (James *et al*, 1996) were transformed with one plasmid derived from pPC86 (GAL4‐activation domain, AD (‘Prey’)) and pPC97 (GAL4‐DNA‐binding domain, BD (‘Bait’); Chevray & Nathans, 1992; Kerssen *et al*, 2006) containing genes encoding proteins of interest. These were selected on YNBG plates (0.17% [w/v] yeast nitrogen base without amino acids, 0.5% [w/v] ammonium sulfate, 2% [w/v] glucose, amino acids according to auxotrophic requirements, pH 6.0) lacking leucine (leu) and tryptophan (trp). Clones were streaked onto YNBG‐trp‐leu (control), YNBG ‐trp‐leu‐his‐ade, and/or YNBG ‐trp‐leu‐his +5 mM 3‐amino triazole (3‐AT) plates and incubated for 3, 7, or 10 days at 30°C, respectively. HIS3 and ADE2 are under the control of GAL1 or GAL2 promoters, respectively. Thus they are only expressed when GAL‐AD and GAL‐BD of the bait and prey proteins are in close proximity due to protein–protein interaction. Nevertheless, ADE2 is a very stringent selection, while HIS3 is not as stringent. Thus, to visualize very weak interactions, adenosine was included in the media to lower the stringency, and 3‐AT (a competitive inhibitor of the HIS3 gene product) was included to inhibit false‐positive growth. This way, the cells can only grow when a large amount of the HIS3 gene product is present (James *et al*, 1996). As a positive control, the interaction of Pex9 with Mls1 was used (preprint: Yifrach *et al*, 2022).

### Glr1 and Pex5 plasmid construction

All cloning reactions were performed by the Restriction‐Free (RF) method (Unger *et al*, 2010). Full‐length yeast *PEX5*, *PEX5 N′* (1–312), and *PEX5 C′* (313–612) were cloned into the expression vector pET28 (Zahradník *et al*, 2019). Yeast *GLR1* was cloned into the first ORF of the expression vector pACYCDuet‐1 (Novagen) including an N‐terminal Flag‐tag followed by a TEV cleavage site.

### Glr1 and Pex5 protein expression

*GLR1* (in *pACYCDut‐FLAG‐GLR1*) and *PEX5* (in *pET28‐PEX5*) were either individually or co‐expressed in *E. coli* BL21(DE3). Expression was performed in LB medium supplemented with the appropriate antibiotics (Kanamycin and/or chloramphenicol). Expression was induced with 200 μM IPTG followed by shaking at 15°C for ~16 h. Cell pellets were stored at −20°C before processing.

### Glr1 and Pex5 protein pull‐down

Cells were lysed by sonication in Tris‐buffered saline (TBS) buffer supplemented with 1 mM phenylmethylsulfonyl fluoride (PMSF) and 1 μl/ml of protease inhibitor cocktail (Set IV, EMD Chemicals, Inc). Protein pull‐down experiments were performed using FLAG Beads (L00432‐10, A2S) according to the manufacturers' recommendations. Proteins were eluted using free FLAG‐peptides (F3290, Sigma). Western blot analysis was performed using THE™ DYKDDDDK Tag Antibody [HRP‐conjugated] (A01428, GenScript) and Monoclonal Anti‐polyHistidine−Peroxidase (A7058, Sigma). Proteins were analyzed on 4–20% SurePAGE precast gels (M00657, GeneScript).

### Metabolite extraction for high‐throughput metabolomics

A target OD_600_ of 1.5 was set for all metabolite extractions for peroxisomal mutant collection experiments with at least two cell doublings occurring between culture inoculation and extraction. For analysis of *AYT1* shown in Fig 4, OD_600_ values between 0 and 1.5 were collected for each of the treatment conditions. Cultivations were performed in a 96‐well format with starting volumes of 1.2 ml per well. OD_600_ values were measured before harvesting cells for extraction. For glucose‐grown samples, cells were grown for 4 h in synthetic defined media as described in the “Yeast growth media” section, including 2% (w/v) glucose. For oleate‐grown samples, cells were first grown in synthetic defined media with a low concentration of glucose (0.1% w/v) for 16 h. Then, cells were transferred to oleate‐containing media (0.2% w/v) and were allowed to grow for additional 2 h before harvesting. Cells were harvested by centrifugation for 1 min at 2254 rcf. After discarding the supernatant, 150 μl of cold extraction solution (40% (v/v) HPLC‐grade acetonitrile (Sigma‐Aldrich: 34998), 40% (v/v) HPLC grade methanol (Sigma‐Aldrich: 34885), 20% (v/v) HPLC‐grade water (Sigma‐Aldrich: 1153331000)) was added to each cell pellet. Extraction was allowed to proceed at −20°C for a duration of 1 h in a covered container. The extracts were exposed to 1 min of centrifugation at 2,254 rcf and 100 μl of the supernatant was taken and transferred into conical 96‐well plates (Huber lab: 7.1058). Plates were sealed (Huber lab: 7.0745) and placed at −80°C until the time of measurement.

### Flow injection time‐of‐flight mass spectrometry for high‐throughput metabolomics

Mass spectrometric measurements were made using an Agilent 6550 Series quadrupole time‐of‐flight mass spectrometer (Agilent) through an adaptation of the method described by Fuhrer *et al* (2011). An Agilent 1100 Series HPLC system (Agilent) was coupled to a Gerstel MPS 3 autosampler (Gerstel) to perform the analysis. A mobile phase flow rate of 0.15 ml/min was used, with the isocratic phase composed of 60:40 (v/v) isopropyl alcohol and water at a buffered pH of 9, with 4 mM ammonium fluoride. Taurocholic acid and Hexakis (1H, 1H, 3H‐tetrafluoropropoxy–phosphazine) within the mobile phase were used to perform online mass axis correction. The instrument was run in high‐resolution (4 GHz) mode, and mass spectra between 50 and 1,000 m/z were collected in negative mode. Raw data files were deposited in the MassIVE repository (https://massive.ucsd.edu/). Peroxisomal deletion library data was deposited with accession code MSV000086773 in the MassIVE database (massive.ucsd.edu), overexpression (*TEF2* promoter) library data was deposited under code MSV000086775, and the focused *AYT1* analysis data was deposited under code MSV000086772.

### Analysis of metabolomics mass spectrometry data

Centroiding of the mass spectrum, merging, and ion annotation was performed as described in Fuhrer *et al* (2011). Metabolites used for ion annotation were drawn from the KEGG *Saccharomyces cerevisiae* metabolite library. Data normalization and analysis were performed using the Pandas package (McKinney, 2010) in Python. For the analysis of peroxisomal mutant collections, temporal drifts, as well as OD_600_ effects in ion intensity were corrected for using a LOWESS and linear regression approach respectively. Outlier samples in terms of OD_600_ at the time of sampling as well as in total ion current were discarded. Z‐score transformations or fold‐change calculations were applied to the normalized data. For the analysis of *AYT1* specifically, the slope of ion intensity with respect to OD_600_ was calculated and average fold‐changes were calculated for *AYT1* mutants relative to WT based on those slopes. GO enrichment analysis was performed as follows: The Manhattan distance between normalized and z‐scored average metabolome profiles was calculated, and a fixed number of clusters were defined for each set of samples (20 groups for loss‐of‐function data, and 18 for *TEF2pr* mediated overexpression) which were clustered by Ward's method. For each cluster, a GO biological process enrichment analysis was then performed using the previously known peroxisomal genes within that cluster as input. GO enrichment was performed using the R package clusterProfiler (Wu *et al*, 2021). The GO term showing the largest enrichment factor (ratio between observed and expected gene ratios) with a Benjamini–Hochberg adjusted *P*‐value of less than 0.05 was then selected for that cluster. The circular dendrogram was then drawn according to the distance and clustering metric described above and the selected GO terms were applied to each cluster to visualize the characteristic GO enrichment for peroxisomal genes within that cluster.

### Growth assay for AYT1 strains

Growth assays were performed with strains based on CB199 (Oeljeklaus *et al*, 2012; Schummer *et al*, 2020) strain (see Dataset EV8). Cells were incubated in 0.3% liquid glucose media, rotating overnight at 30°C. Then, a total of 2 O.D_600_ of cells were centrifuged for 3 min at 3,000 *g*, and pellets were inoculated in 10 ml of either SD (6.7 g/l yeast nitrogen base with ammonium sulfate and 2% glucose), or S(MSG)‐oleate (6.7 g/l yeast nitrogen base without ammonium sulfate, with 1 g/l monosodium glutamate, 0.1% oleate, 0.1% yeast extract, and 0.05% Tween‐40). Both media contained a complete amino acid mix. Before measuring the O.D_600_ in oleate, 1 ml of cells was taken, cells were washed twice with water and resuspended in 1 ml water.

### Lipid extraction for high‐throughput lipidomics

The indicated yeast strains were cultivated in synthetic defined media supplemented with amino acids and G418 (deletion strains) or NAT (overexpression strains) antibiotics. For glucose‐grown samples, cells were grown for 4 h in synthetic defined media as described in the “Yeast growth media” section, including 2% (w/v) glucose. For oleate‐grown samples, cells were first grown in synthetic defined media with a low concentration of glucose (0.1% w/v) for 16 h. Then, cells were transferred to oleate‐containing media (0.2% w/v) and were allowed to grow for additional 2 h before harvesting. Yeasts were harvested by centrifugation at 2,254 rcf for 1 min and the supernatant was discarded. Lipid extraction was performed as described previously (Pellegrino *et al*, 2014) with some modifications. To 20 μl of the sample, 1 ml of a mixture of methanol: IPA 1:1 (v/v/v) was added. The mixture was fortified with the SPLASH mix of internal standards (Avanti Lipids). After brief vortexing, the samples were continuously mixed in a Thermomixer (Eppendorf) at 25°C (950 rpm, 30 min). Protein precipitation was obtained after centrifugation for 10 min, 16,000 *g*, 25°C. The single‐phase supernatant was collected, dried under N2, and stored at −20°C until analysis. Before Analysis, the dried lipids were re‐dissolved in 100 μl MeOH:Isopropanol (1:1 v/v).

### Liquid chromatography and mass spectrometry for lipidomic analysis

Liquid chromatography was done as described previously (Cajka & Fiehn, 2016) with some modifications. The lipids were separated using C18 reverse‐phase chromatography. Vanquish LC pump (Thermo Scientific) was used with the following mobile phases; (i) Acetonitrile:water (6:4) with 10 mM ammonium acetate and 0.1% formic acid and (ii) Isopropanol:Acetonitrile (9:1) with 10 mM ammonium acetate and 0.1% formic acid. The Acquity BEH column (Waters) with the dimensions 100 mm × 2.1 mm × 1.7 μm (length × internal diameter × particle diameter) was used. The following gradient was used with a flow rate of 1.2 ml/min; 0.0–0.29 min (isocratic 30%B), 0.29–0.37 min (ramp 30–48% B), 0.370–1.64 min (ramp 48–82%B), 1.6–1.72 min (ramp 82–99%), 1.72–1.79 min (isocratic 100%B), 1.79–1.81 min (ramp 100–30% B), and 1.81–2.24 min (isocratic 30%B). The liquid chromatography was coupled to a hybrid quadrupole‐orbitrap mass spectrometer (Q‐Exactive HF‐X, Thermo Scientific). A full scan acquisition in negative and positive ESI was used. A full scan was used scanning between 200 and 2,000 m/z at a resolution of 60,000 and AGC target 1e6. The maximum injection time was 100 ms.

### Analysis of lipidomics mass spectrometry data

Lipid identification was achieved using the following criteria: (i) high accuracy and resolution with accuracy within m/z within 5 ppm shift from the predicted mass, (ii) Isotopic pattern fitting to expected isotopic distribution and (iii) retention order compared to an in‐house database. Quantification was done using single‐point calibration against the SPLASH internal standard (Avanti Lipids). Mass spectrometric data analysis was performed in Compound Discoverer 3.1 (Thermo Scientific) for peak picking, integration, and annotation. Class enrichment analysis was performed through the application of a hypergeometric test for enrichment of significantly changing lipids compared to the representation of those classes within the set of annotated lipids. Raw mass spectra were deposited with accession code MSV000086777 in the MassIVE database (massive.ucsd.edu).

### β‐Oxidation activity in peroxisomes

β‐Oxidation assays in intact cells were performed as described previously (van Roermund *et al*, 1998) with slight modifications. Cells were grown overnight in media containing 0.5% glucose. The β‐oxidation capacity was measured in 50 mM MES, pH 6.0 supplemented with 10 μM 1‐^14^C‐octanoate (C8:0) or 10 μM 1‐^14^C oleate (C18:1). Subsequently, [^14^C]CO_2_ was trapped with 2 M NaOH and acid‐soluble counts (ASP) were used to quantify the rate of fatty acid oxidation. Results are presented as percentages relative to the rate of oxidation of control cells.

### Subcellular fractionation

Subcellular fractionation was performed as described by Van der Leij *et al* (1992). Briefly, a protoplast‐free and a nuclei‐free organellar pellet was loaded onto a 15–35% continuous Nycodenz gradient (111 ml), with a cushion of 50% Nycodenz (1 ml), dissolved in buffer A (5 mM MES pH 6.0, 1 mM EDTA, 1 mM KC1 and 8.5% (w/v) sucrose). After centrifugation for 2.5 h in a vertical rotor (MSE 8 × 35) at 29,000 *g* at 4°C, the gradient was unloaded from the bottom, yielding 12 fractions.

### Enzymatic activity of Fum1, Fox2, and Glr1 in subcellular fractions

The 3‐hydroxyacyl‐CoA dehydrogenase activity of Fox2 was measured as described by Wanders *et al* (1990). Fumarase was measured as described by van Roermund *et al* (1999). Glutathione reductase activity was measured at 37°C by monitoring the absorption at 340 nm over a period of 5 min using a reaction medium with the following components: 1 mM EDTA, 50 mM TRIS buffer pH 7.2, 0.1% (v/v) Triton X‐100, 0.1 mM NADPH, 5 mM GSSG and the sample to be analyzed. Since the reaction in the absence of enzyme does take place at an appreciable rate, the change in absorbance of a solution containing no sample was also recorded and subtracted.

### Western blot for Fbp1‐HA strains

Cells were grown in glucose‐containing media with appropriate selections overnight at 30°C. Then, cells were diluted to 0.2 OD_600_ in YPD (2% Peptone + 1% Yeast Extract (Formedium) and 2% glucose) and grown for additional 3 h. At mid‐log (0.5–0.8 OD_600_), a total of 3 OD_600_ cells were taken, washed with double‐distilled water, and the pellet was snap‐frozen in liquid nitrogen. In parallel, an additional 3 OD_600_ of cells were taken, washed with double‐distilled water, and inoculated in an S‐ethanol medium. Cells were incubated for 2 h at 30°C and then washed with water, pelleted, and snap‐frozen in liquid nitrogen. Samples were stored at −80°C until protein extraction.

For protein extraction, pellets were resuspended in urea lysis buffer (8 M Urea, 50 mM Tris pH 7.5, and 1:200 freshly added protease inhibitor cocktail (Merck)). One hundred microlitre acid‐washed glass beads were added, and cells were broken using a bead beater machine, for 10 min at 4°C. Then, each sample was added with 2.5% SDS and incubated at 45°C for 15 min. Samples were centrifuged at 3,000 *g*, 16°C for 10 min, and supernatants were transferred to new tubes for additional centrifugation at max speed, 16°C, for 5 min. Supernatants were collected and mixed with loading buffer (60 mM Tris pH 6.8, 10% glycerol, Orange G dye (Sigma), and 100 mM fresh DTT). Before loading on SDS‐PAGE, samples were incubated again at 45°C for 15 min. Samples ran at a constant voltage of 80 and then transferred to a nitrocellulose membrane using a semi‐dry Bio‐Rad transfer machine. Antibodies used were anti‐HA (BioLegend, monoclonal antibody raised in mouse, 1:1,000 dilution) for Fbp1‐HA, and Actin (Abcam, polyclonal antibody raised in rabbit, 1:1,000 dilution).

### Western blot on *NOP1pr*‐GFP newly identified peroxisomal proteins

Cells were grown in glucose‐containing media as described in the main text. Whole‐cell protein extraction was performed by either the Urea protein extraction method (described in Fbp1‐HA western blot method) or the NaOH protein extraction method. In short, for extracting proteins using the NaOH method, 3 OD_600_ of cells were incubated in 0.2 M of NaOH for 5 min, at room temperature. Following centrifugation, pellets were re‐suspended with SDS‐sample buffer (60 mM Tris pH 6.8, 2% SDS, 10% glycerol, 200 mM DTT, and 0.2 mM freshly added PMSF), and were boiled at 95°C for 5 min. Before loading on an acrylamide gel, samples were centrifuged at max speed for 3 min. Samples ran at a constant voltage of 80 and then transferred to a nitrocellulose membrane using a semi‐dry Bio‐Rad transfer machine. The antibody used was anti‐GFP (Abcam, polyclonal antibody raised in rabbit, 1:2,000 dilution).

## Author contributions

**Eden Yifrach:** Investigation; writing – original draft; writing – review and editing. **Duncan Holbrook-Smith**: Investigation. **Jérôme Bürgi**: Investigation. **Alaa Othman**: Investigation. **Miriam Eisenstein**: Designing, executing and writing the molecular dynamics computations and analyses. **Carlo W T Van Roermund**: Investigation. **Wouter Visser**: Investigation. **Asa Tirosh**: Investigation. **Markus Rudowitz**: Investigation. **Chen Bibi**: Investigation. **Shahar Galor**: Investigation. **Uri Weill:** Methodology. **Amir Fadel**: Investigation. **Yoav Peleg:** Supervision. **Ralf Erdmann:** Supervision; funding acquisition. **Hans Waterham:** Supervision. **Ronald J A Wanders:** Supervision. **Matthias Wilmanns:** Supervision. **Nicola Zamboni:** Supervision. **Maya Schuldiner:** Conceptualization; supervision; funding acquisition; writing – original draft; writing – review and editing. **Einat Zalckvar:** Conceptualization; supervision; writing – original draft; writing – review and editing.

## Disclosure and competing interests statement

The authors declare that they have no conflict of interest. MS is an editorial advisory board member. This has no bearing on the editorial consideration of this article for publication.

## Supporting information



Appendix

Dataset EV1

Dataset EV2

Dataset EV3

Dataset EV4

Dataset EV5

Dataset EV6

Dataset EV7

Dataset EV8

Dataset EV9

Review Process File

## Data Availability

The datasets produced in this study are available in the following database: Imaging datasets: BioImage Archive S‐BIAD532 (https://www.ebi.ac.uk/biostudies/studies/S‐BIAD532)
