# Supplementary material for: Systematic multi‐level analysis of an organelle proteome reveals new peroxisomal functions
Source: Mol Syst Biol. 2022 Sep 27;18(9):e11186. doi: 10.15252/msb.202211186 (PMC9513677; doi:10.15252/msb.202211186)
Supplement: Supplementary file 1 — Appendix [file MSB-18-e11186-s011.pdf]

Appendix for

Systematic multi-level analysis of an organelle proteome reveals new peroxisomal functions

Eden Yifrach[1], Duncan Holbrook-Smith[2], Jérôme Bürgi[3], Alaa Othman[2], Miriam Eisenstein[1], Carlo W.T Van Roermund[5], Wouter Visser[5], Asa Tirosh[6], Markus Rudowitz[7], Chen Bibi[1], Shahar Galor[1], Uri Weill[1], Amir Fadel[1], Yoav Peleg[6], Ralf Erdmann[7], Hans R Waterham[5], Ronald J A Wanders[5], Matthias Wilmanns[3,4], Nicola Zamboni[2], Maya Schuldiner[1*] and Einat Zalckvar[1*]

Table of content

Appendix figure S1: Page 2

Appendix figure S2: Page 3

Appendix figure S3: Page 4

Appendix figure S4: Page 5

Appendix figure S5: Page 6

Appendix figure S6: Pages 7-8

Appendix figure S7: Page 9

Appendix figure S8: Page 10

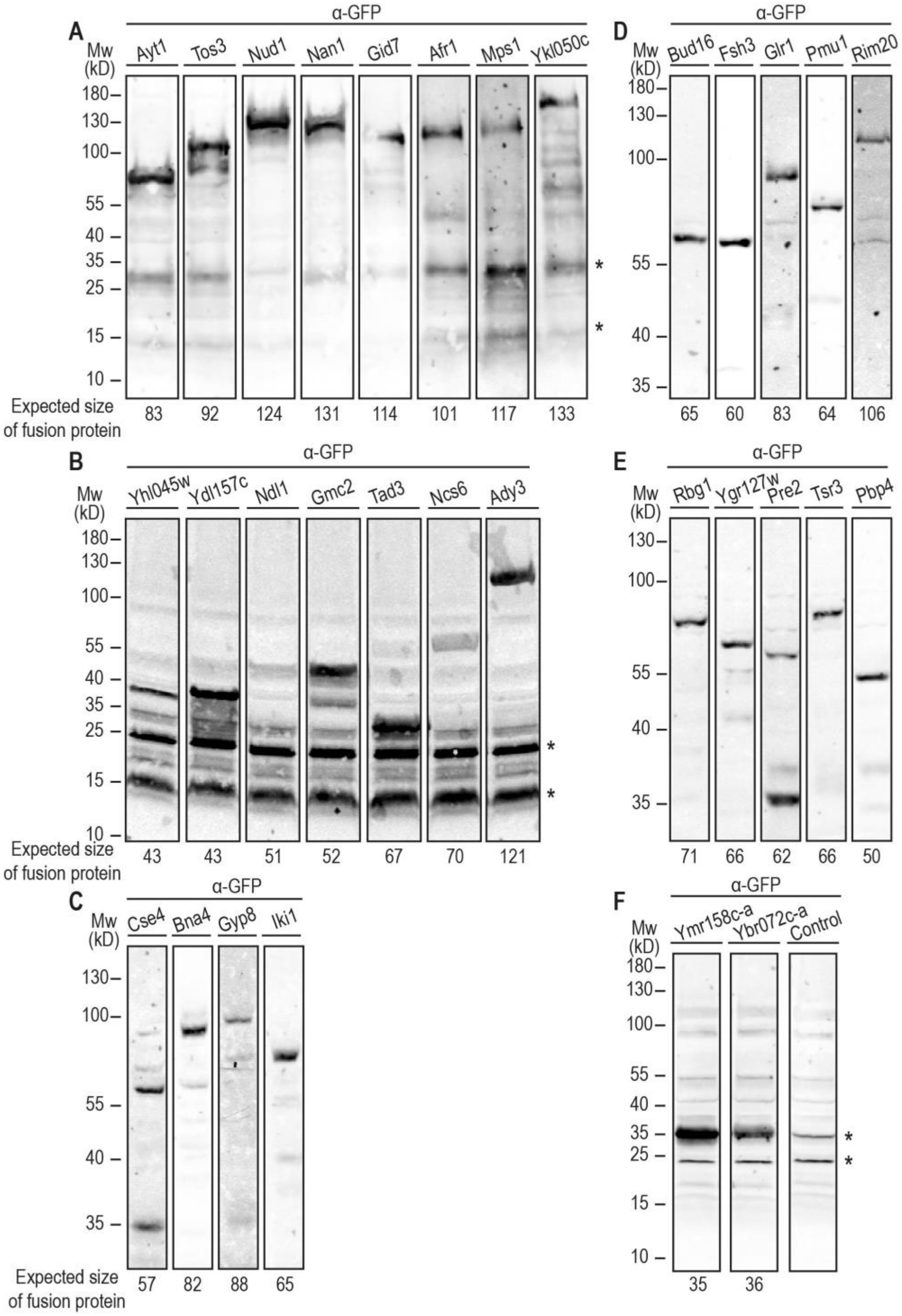

Appendix Figure S1. Western blot analysis of candidate peroxisomal proteins ascertains correct genomic integration. Protein samples of strains expressing each N’ GFP candidate peroxisomal protein were immunodecorated with an antibody against the GFP tag and analyzed for their expected molecular weight. Asterisks indicate non-specific bands. Tyc1 and Sws2 were not detected. Panel (A) shows an analysis of proteins that were extracted by Urea. Panels (B)-(F) show analysis of proteins that were extracted by NaOH. Data information: Pre-cast 4-20% acrylamide gels (Bio-Rad) were used.

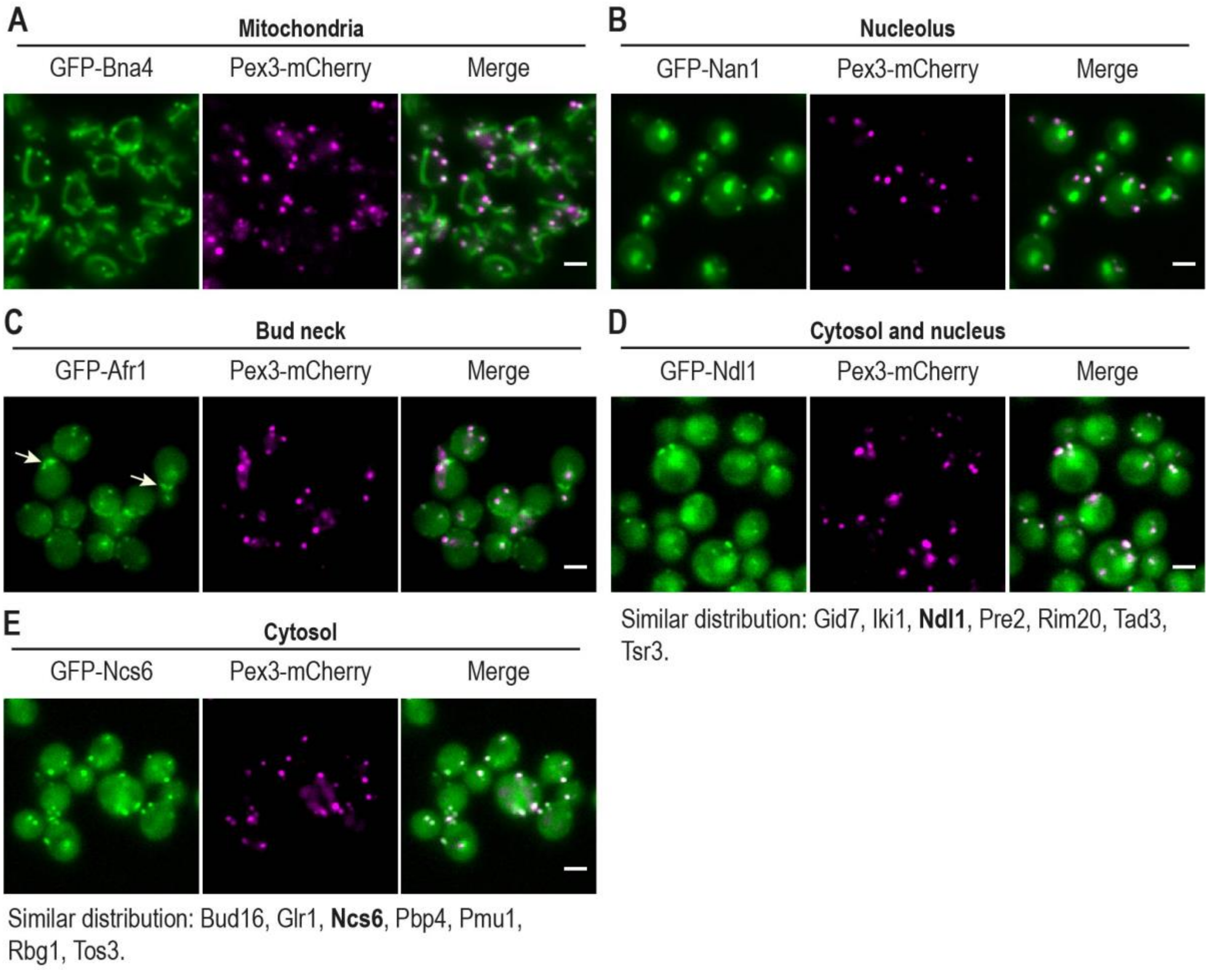

Appendix Figure S2. Image analysis of the newly-identified peroxisomal proteins indicates that half of them have multiple cellular localizations. (A) GFP-Bna4 is dually localized to mitochondria and peroxisomes. (B) GFP-Nan1 is dually localized to nucleolus and peroxisomes. (C) GFP-Afr1 is localized to the bud neck (white arrows) and peroxisomes. (D) Seven proteins are localized to the cytosol, nucleus, and peroxisomes. GFP-Ndl1 is an example from this group. (E) Seven proteins are dually localized to the cytosol and peroxisomes. GFP-Ncs6 is an example from this group. Data information: Proteins with the same distribution are listed below each image when the protein in bold is the one presented. For all micrographs, a single focal plane is shown and the scale bar is 5 µm. The analysis considered proteins as dual localized only when their tagged protein was visualized in at least one localization similar to its ‘cellular compartment’ annotation in the *Saccharomyces* Genome Database (SGD) (Cherry et al., 2012) (see also Dataset EV1).

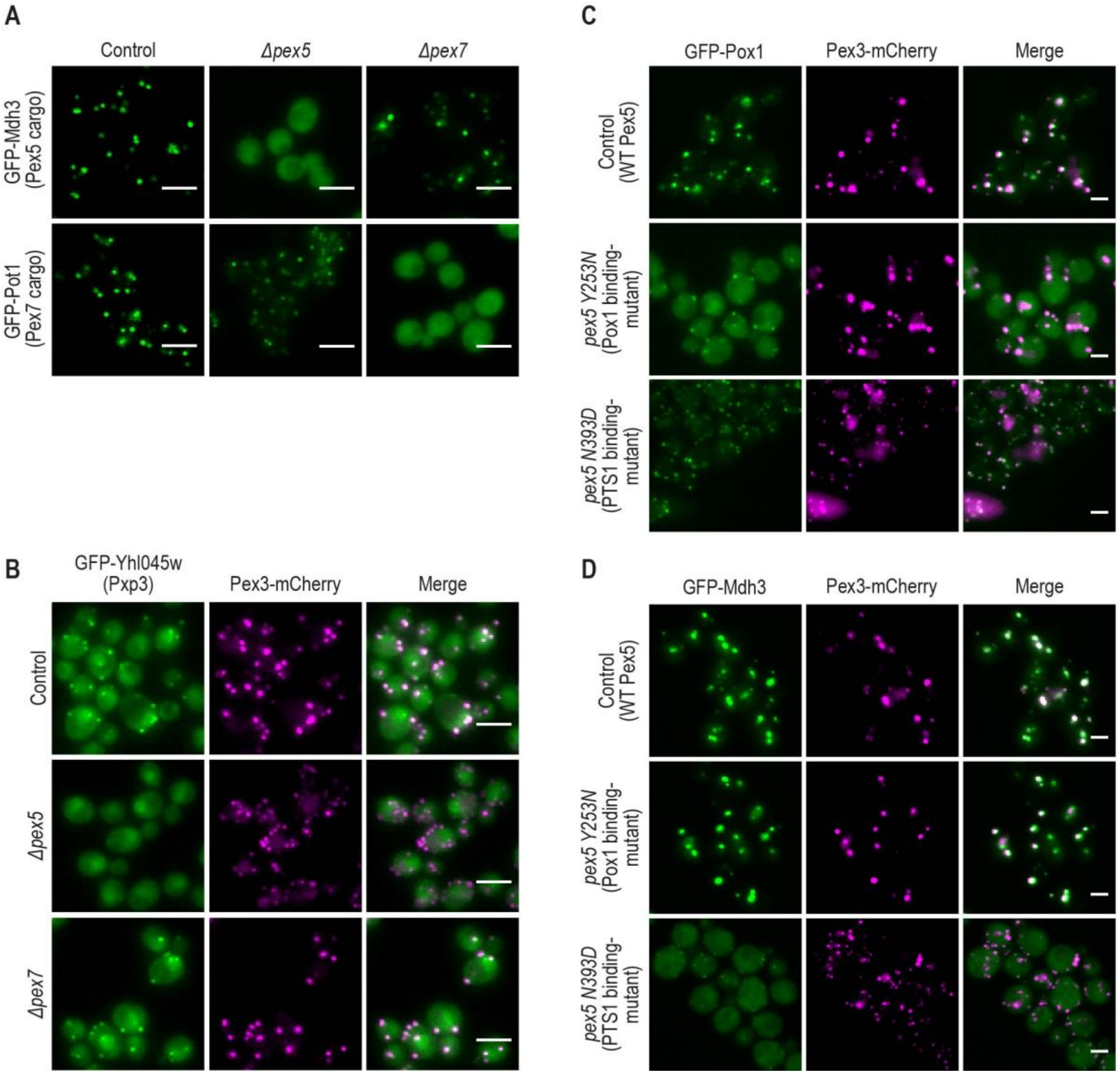

Appendix Figure S3. Verification of functional targeting assays using known cargo proteins. (A) Mdh3 (a Pex5 cargo protein) and Pot1 (a Pex7 cargo protein) were utilized to show expected dependencies on their specific targeting factor using fluorescence microscopy. (B) GFP-Yhl045w (Pxp3), a newly-identified peroxisomal matrix protein, represents a case for targeting dependency on Pex5, but not on Pex7. (C) GFP-Pox1 (a Pex5 cargo protein that interacts with Pex5 in a PTS1-independent manner) was used to show expected dependency upon $Pex5_{Y253N}$ point mutation. (D) GFP-Mdh3 (a known PTS1 protein) was used to show targeting dependency on $Pex5_{N393D}$ (a mutation in the PTS1-binding site). Data information: For all micrographs, a single focal plane is shown. The scale bar is 5 µm.

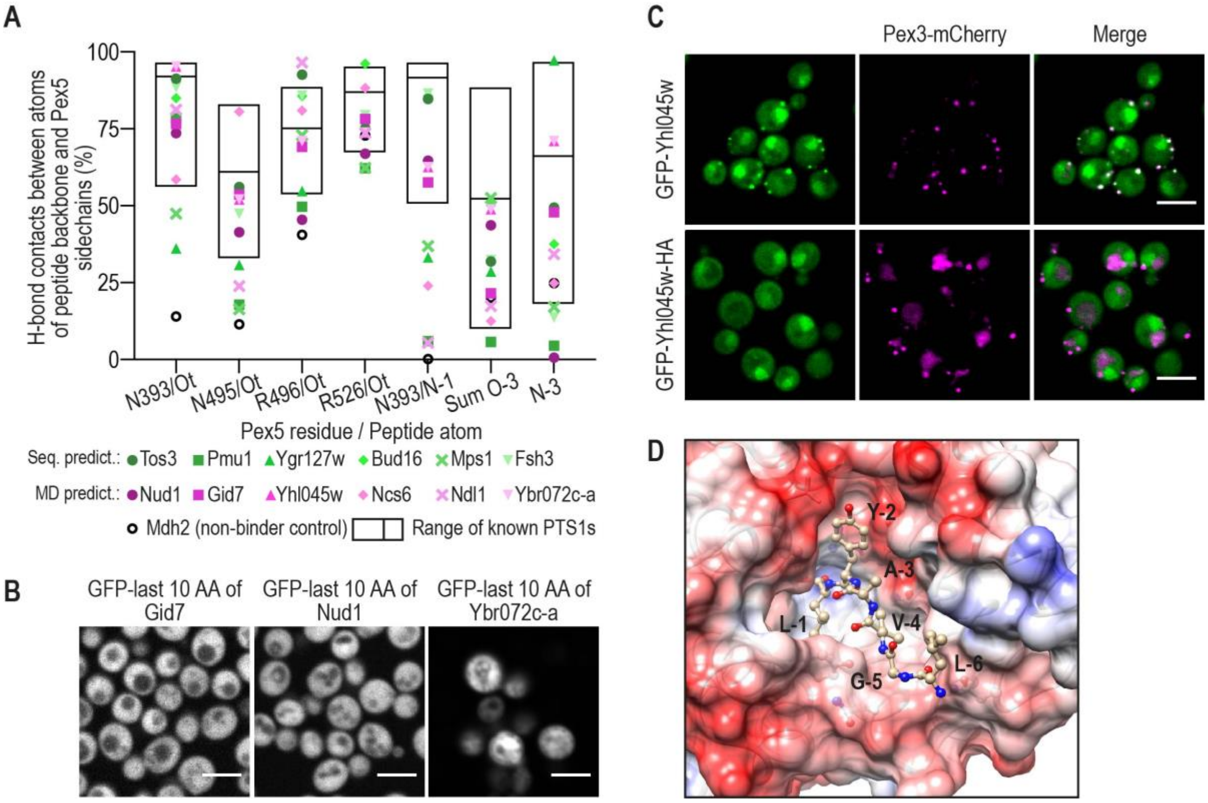

Appendix Figure S4. Characterization of unique PTS1 motifs. (A) Predictions for identifying potential new PTS1 motifs were made by molecular dynamic (MD) simulations. The likelihood of stable binding between Pex5 and the backbone atoms at the C’ amino acids -1 and -3 of the new, putative, cargo proteins was compared to the stability of hydrogen bonds of known PTS1 cargo proteins to Pex5 (Dataset EV3). The ranges of hydrogen bond stabilities for the 22 known PTS1 cargos are presented by box plots; the black lines represent averages. MD results for potential Pex5 cargos previously predicted based on their sequence are presented in green shades. Six proteins predicted by the MD analysis as cargos are presented in magenta shades. Mdh2 was used as a known control for no Pex5 binding. (B) The peroxisomal targeting ability of the predicted motifs was examined by fusing the last 10 amino acids of Gid7, Nud1, and Ybr072c-a to the C’ of GFP, integrating the construct into an inert locus in the yeast genome, and imaging. Most motifs, except for the Yhl045w (Pxp3) motif (Fig. 3C), were unable to target GFP to peroxisomes. (C) Blocking the C’ of GFP-Yhl045w (Pxp3) with an HA tag demonstrates that Yhl045w (Pxp3) C’ is necessary for targeting to peroxisomes. (D) A representative frame from the MD simulations for the Pex5/Pxp3 complex showing the unique Pxp3 PTS1 motif nicely fits into the PTS1-binding pocket of Pex5. The side chain of tyrosine (Y) in position -2 of Pxp3 can be accommodated in a shallow, mildly negatively charged depression within the binding cavity, making numerous contacts including water-mediated contacts that involve the OH group. Data information: For all micrographs, a single focal plane is shown. The scale bar is 5 µm.

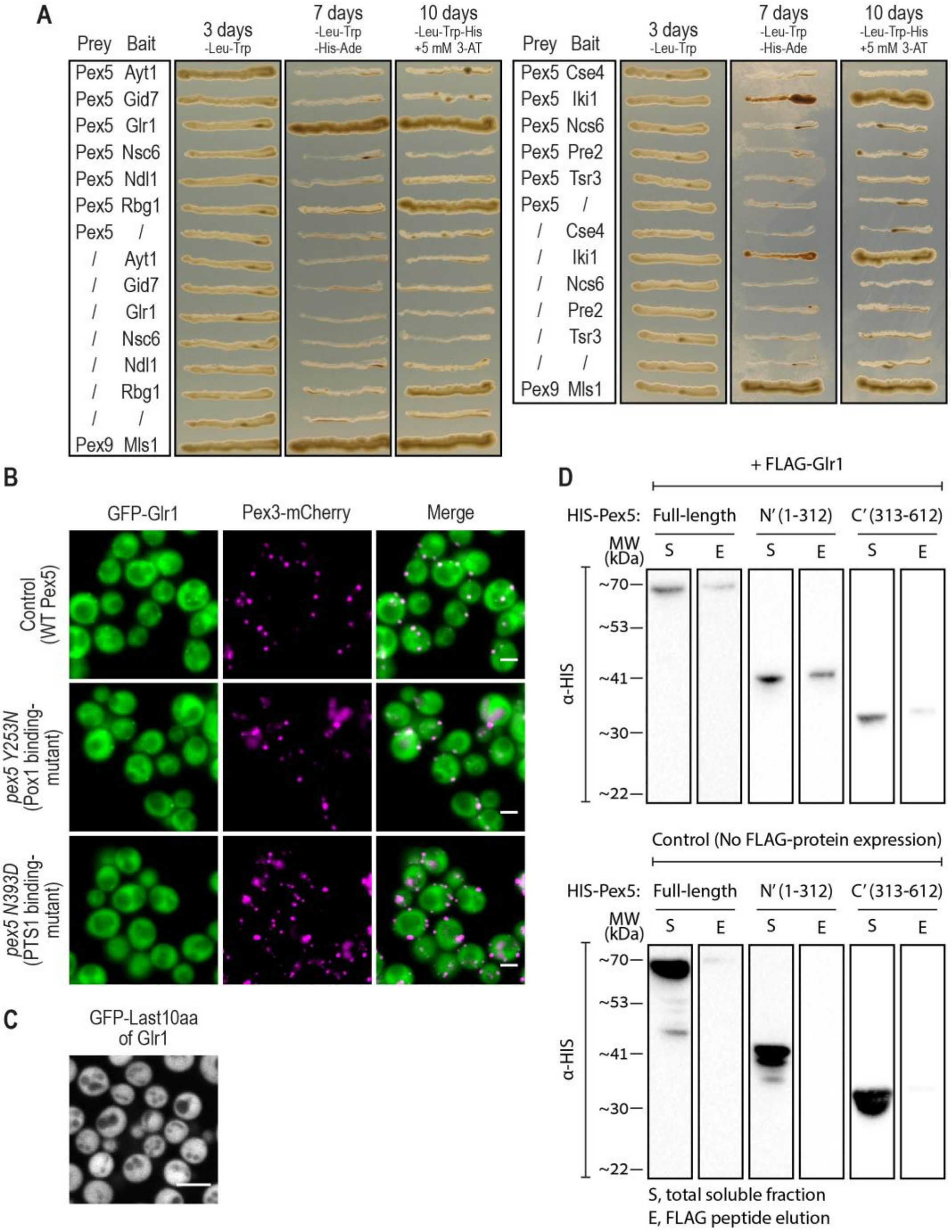

Appendix Figure S5. Glr1 is dependent on the PTS1 targeting route and binds Pex5 N' domain. (A) Yeast-2-hybrid assay for ten different newly-identified peroxisomal proteins shows that Glr1 binds Pex5. (B) GFP-Glr1 peroxisomal localization was abolished upon point mutation in the PTS1-binding domain of Pex5 ($Pex5_{N393D}$). (C) A strain expressing the last ten amino acids of Glr1 fused to a GFP at its C' shows that they are not sufficient to target GFP to peroxisomes, hence Glr1 doesn't contain a PTS1 motif. (D) *In vitro* pull-down assays and western blot analysis show full-length HIS-Pex5 and HIS-Pex5 N' domain co-eluted with FLAG-Glr1, which indicate for a direct and specific interaction between them. The pull-down at the bottom shows that the full-length, N' and C' HIS-Pex5 do not interact non-specifically with the FLAG beads. Data information: S- Total soluble fraction, E- Elution fraction. For all micrographs, a single focal plane is shown. The scale bar is 5 µm.

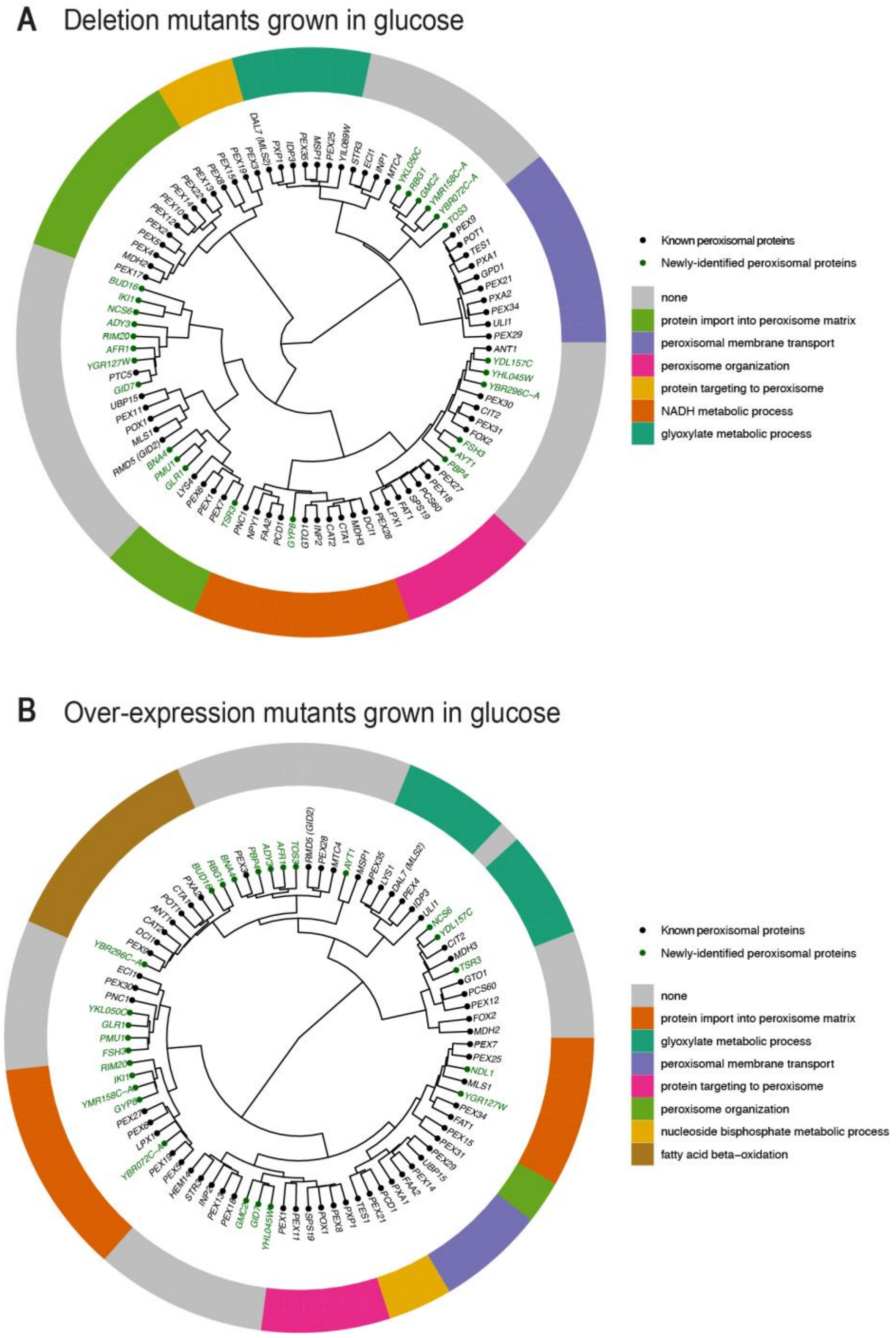
A Deletion mutants grown in glucose
Known peroxisomal proteins
Newly-identified peroxisomal proteins
none
protein import into peroxisome matrix
peroxisomal membrane transport
peroxisome organization
protein targeting to peroxisome
NADH metabolic process
glyoxylate metabolic process
B Over-expression mutants grown in glucose
Known peroxisomal proteins
Newly-identified peroxisomal proteins
none
protein import into peroxisome matrix
glyoxylate metabolic process
peroxisomal membrane transport
protein targeting to peroxisome
peroxisome organization
nucleoside bisphosphate metabolic process
fatty acid beta–oxidation

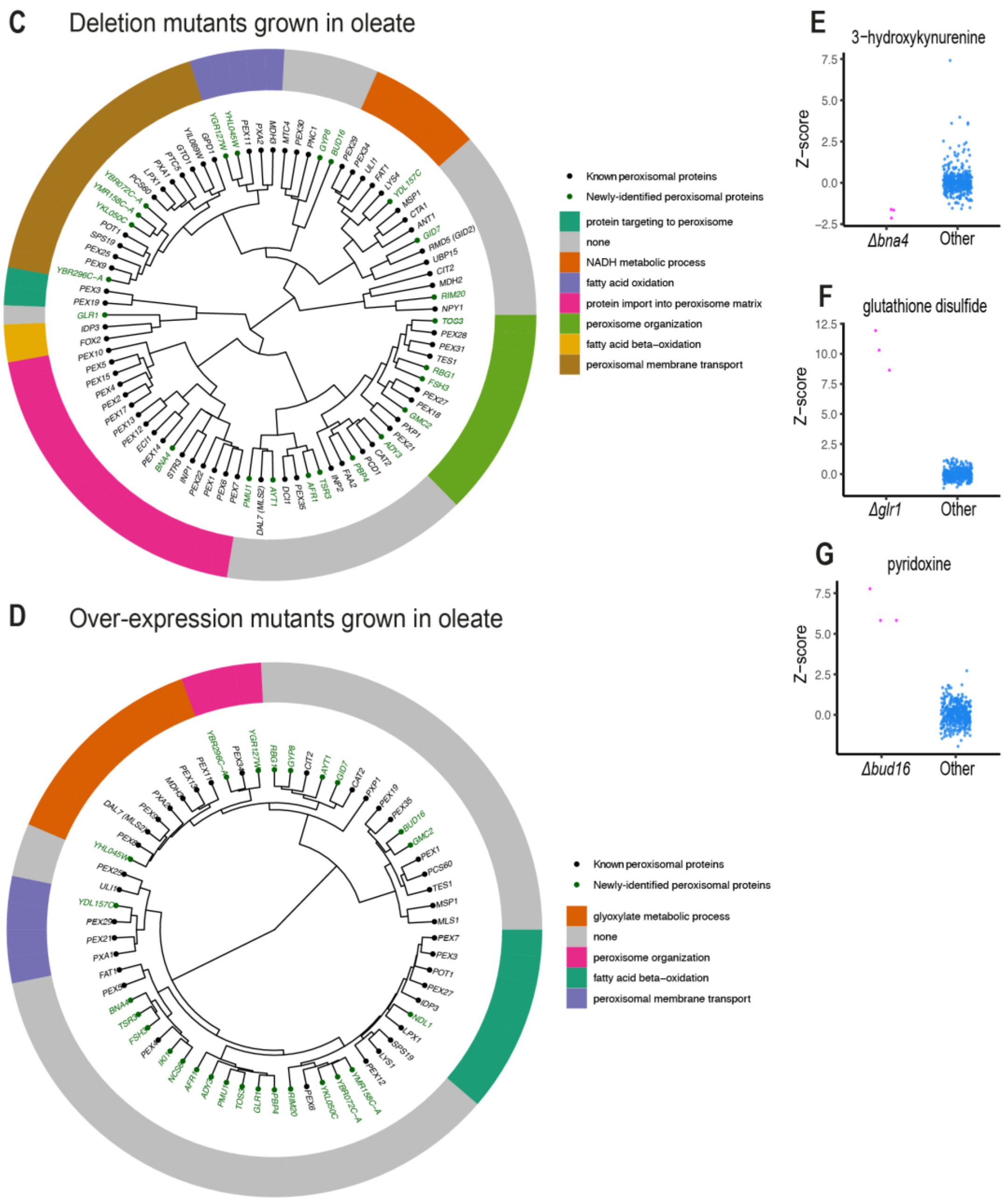

Appendix Figure S6. The hierarchical relationships between metabolomic fingerprints of different peroxisomal mutants. A circular dendrogram shows the Manhattan distance between the average z-scored metabolome profiles of the indicated strains with clustering by Ward's method. The most enriched GO biological process for the peroxisomal genes in each cluster is indicated only for those where a significant enrichment is observed (Benjamini-Hochberg adjusted p-valued < 0.05). (A) Deletion mutants grown in glucose. (B) Over-expression mutants (*TEF2* promoter) grown in glucose. (C) Deletion mutants grown in Oleate. (D) Over-expression mutants (*TEF2* promoter) grown in Oleate. (E) The established activity of the Kynurenine 3-monooxygenase, Bna4, was detected by the metabolomic analysis of its deletion strain (reduction in 3-hydroxykynurenine). (F) The known activity of the glutathione oxidoreductase, Glr1, was captured by metabolomics, with elevation in glutathione disulfide molecules upon deletion of Glr1. (G) The putative role of Bud16 as a pyridoxal kinase was strengthened by the metabolomics data, showing *Δbud16* had elevated levels of pyridoxine.

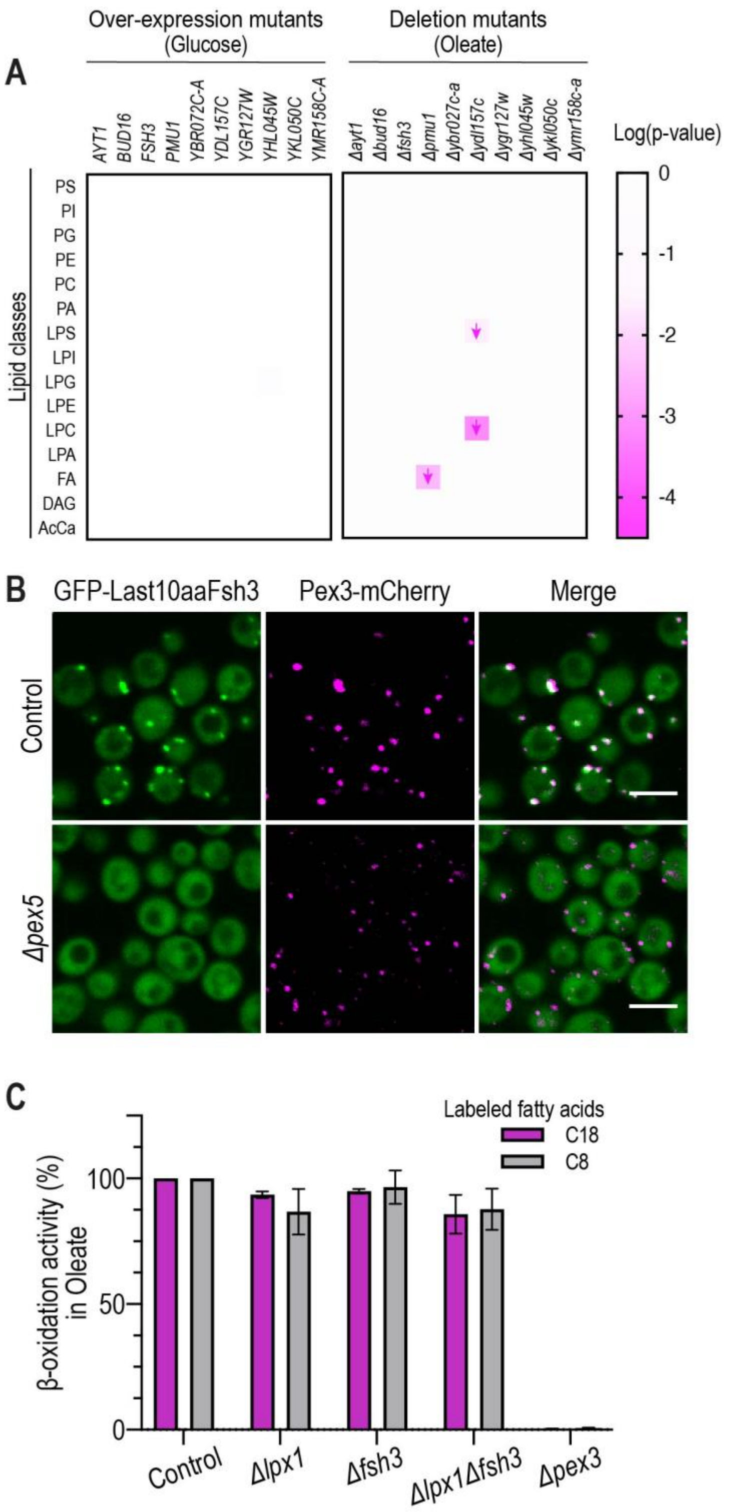

Appendix Figure S7. Functional assays for identifying mutants that affect lipid metabolism. (A) Lipidomic analysis of peroxisomal deletion or over-expression mutants grown in glucose- or oleate-containing media are summarized in a heatmap. Arrows indicate directionality of fold-change. (B) The last 10 amino acids of Fsh3 were sufficient to support the targeting of GFP to peroxisomes in a Pex5-dependent manner, demonstrating Fsh3 has a unique PTS1 motif. The scale bar is 5 µm. (C) A β-oxidation activity assay of *Δlpx1, Δfsh3*, and *Δlpx1Δfsh3* strains supplemented with labeled 8 carbon- or 18 carbon- fatty acids was measured following growth in oleate-containing media, showing no difference in β-oxidation activity between strains.

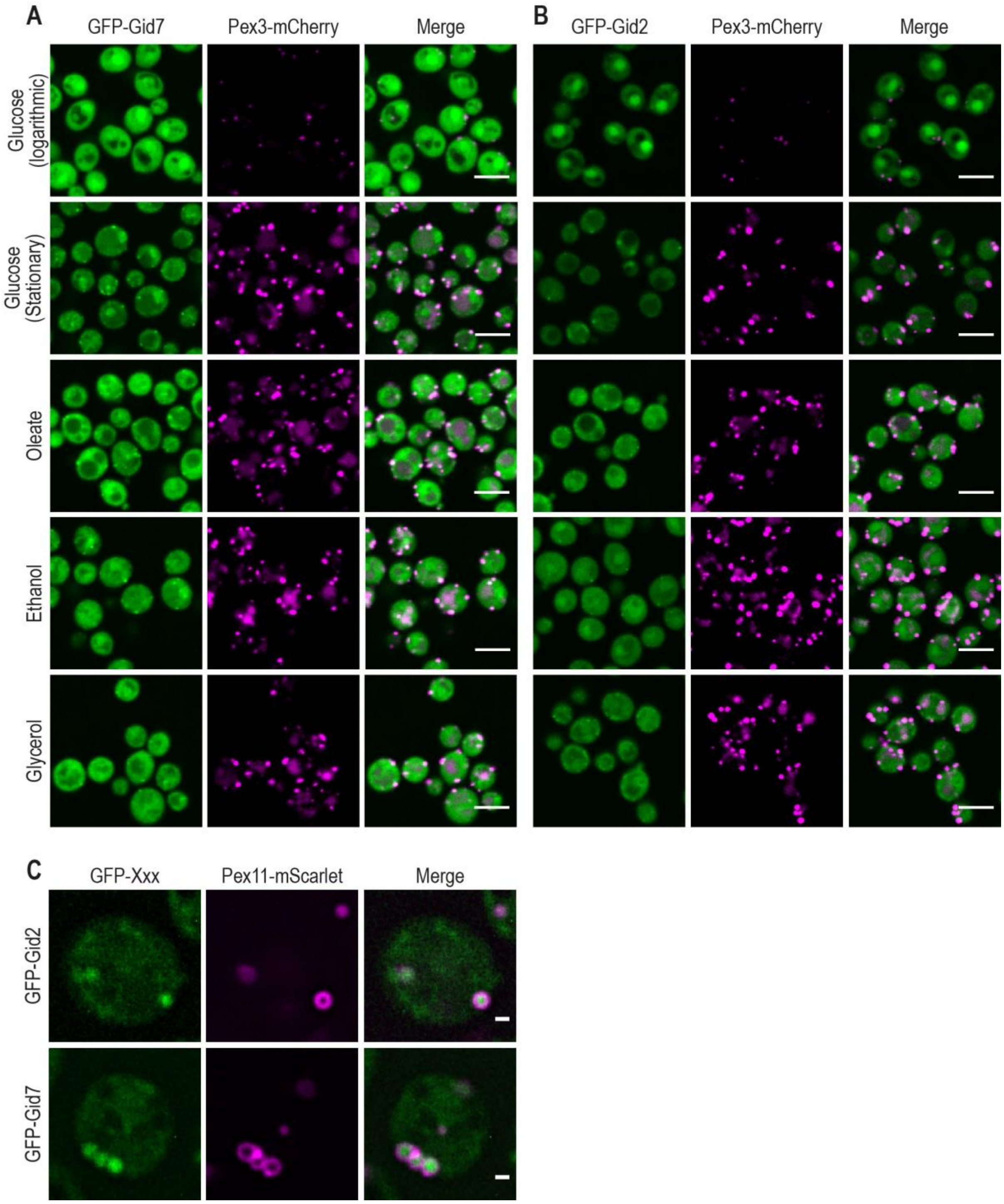

Appendix Figure S8. GID complex subunits Gid7 and Gid2 are targeted to the peroxisomal matrix only in gluconeogenic conditions. Micrographs of strains expressing (A) GFP-Gid7 and (B) GFP-Gid2 show that they localize to the cytosol when cells are grown in media supplemented with glucose. However, they partially localize to peroxisomes when cells are grown in media without glucose. Glucose (logarithmic) and Glucose (stationary) images for GFP-Gid7 are identical to the main figure 5C. The scale bar is 5 µm. (C) High-resolution imaging shows both GFP-Gid2 and GFP-Gid7 localize to the peroxisomal matrix during growth in oleate. The scale bar is 500 nm. Data information: For all micrographs, a single focal plane is shown.
